# *GNAS* knockout potentiates HDAC3 inhibition through viral mimicry-related interferon responses in lymphoma

**DOI:** 10.1038/s41375-024-02325-4

**Published:** 2024-08-08

**Authors:** Michael Y. He, Kit I. Tong, Ting Liu, Ryder Whittaker Hawkins, Victoria Shelton, Yong Zeng, Mehran Bakhtiari, Yufeng Xiao, Guangrong Zheng, Ali Sakhdari, Lin Yang, Wenxi Xu, David G. Brooks, Rob C. Laister, Housheng Hansen He, Robert Kridel

**Affiliations:** 1 Princess Margaret Cancer Centre, University Health Network, Toronto, ON Canada; 2 Department of Immunology, University of Toronto, Toronto, ON Canada; 3 Department of Medicinal Chemistry, College of Pharmacy, University of Florida, Gainesville, FL USA; 4 Laboratory Medicine and Pathobiology, University Health Network, Toronto, ON Canada; 5 Department of Medical Biophysics, University of Toronto, Toronto, ON Canada; 6 Institute of Medical Science, University of Toronto, Toronto, ON Canada; 7 Department of Medicine, University of Toronto, Toronto, ON Canada; 8 Present Address: Cell Biology Program, Hospital for Sick Children, Toronto, ON Canada

**Keywords:** B-cell lymphoma, Targeted therapies, Cancer therapeutic resistance, Translational research

## Abstract

Despite selective HDAC3 inhibition showing promise in a subset of lymphomas with *CREBBP* mutations, wild-type tumors generally exhibit resistance. Here, using unbiased genome-wide CRISPR screening, we identify *GNAS* knockout (KO) as a sensitizer of resistant lymphoma cells to HDAC3 inhibition. Mechanistically, *GNAS* KO-induced sensitization is independent of the canonical G-protein activities but unexpectedly mediated by viral mimicry-related interferon (IFN) responses, characterized by TBK1 and IRF3 activation, double-stranded RNA formation, and transposable element (TE) expression. *GNAS* KO additionally synergizes with HDAC3 inhibition to enhance CD8^+^ T cell-induced cytotoxicity. Moreover, we observe in human lymphoma patients that low *GNAS* expression is associated with high baseline TE expression and upregulated IFN signaling and shares common disrupted biological activities with *GNAS* KO in histone modification, mRNA processing, and transcriptional regulation. Collectively, our findings establish an unprecedented link between HDAC3 inhibition and viral mimicry in lymphoma. We suggest low *GNAS* expression as a potential biomarker that reflects viral mimicry priming for enhanced response to HDAC3 inhibition in the clinical treatment of lymphoma, especially the *CREBBP* wild-type cases.

## Introduction

Diffuse large B-cell lymphoma (DLBCL) and follicular lymphoma are the two most common subtypes of non-Hodgkin lymphoma, characterized by highly heterogeneous biological, pathological, and clinical features. Challenges remain in the clinical management of both diseases—follicular lymphoma is largely considered incurable, and up to 40% of all DLBCL patients experience treatment failure after standard immunochemotherapy R-CHOP with only a subset of patients successfully salvaged with stem cell transplantation or immunotherapy [1, 2]. Novel treatment options are therefore urgently required. By targeting the critical lymphoma hallmark of epigenetic deregulation, epigenetic therapy has shown promise via induction of anti-tumor effects including restoration of immune surveillance and growth inhibition [3, 4].

Reduced acetylation at histone 3 lysine 27 (H3K27ac) represents a pathogenetic hallmark of B-cell lymphoma, leading to disrupted enhancer activity and promoting tumorigenesis [5, 6]. This epigenetic defect results from highly recurrent mutations in epigenetic regulators such as the histone acetyltransferase CREBBP. Somatic *CREBBP* mutations cause functional loss of the acetyltransferase activity and are frequent in DLBCL (6–22%) and follicular lymphoma (31–68%), with more than 40% of the cases in germinal center B-cell-like (GCB)-DLBCL and 17% in activated B-cell-like (ABC)-DLBCL [7–9]. Importantly, CREBBP inactivation results in unopposed deacetylation at H3K27 in the enhancer regions of genes responsible for B-cell signaling and immune responses [6], leading to abnormal gene silencing and lymphomagenesis.

Histone deacetylase 3 (HDAC3) is recruited by the transcription repressor BCL6 to form a deacetylation complex with SMRT and NCoR to counteract the activity of CREBBP for gene enhancer regulation, which maintains the unopposed deacetylation state in *CREBBP*-mutant lymphoma [5, 6, 10]. Based on this rationale, HDAC3 loss-of-function or selective HDAC3 inhibition has been recently shown to induce strong anti-lymphoma effects by increasing H3K27ac in *CREBBP*-mutant lymphoma while those effects are modest in *CREBBP* wild-type tumors [6, 11]. Mechanistically, HDAC3 inhibition has been demonstrated to derepress BCL6-repressed interferon (IFN) responses and antigen-presenting genes and enhance the killing effects of tumor-infiltrating lymphocytes [11]. Combining HDAC3 inhibition with PD-L1 blockade further results in synergistic effects [11, 12], suggesting integrated anti-tumor immunomodulating effects of epigenetic agents and immunotherapy. Given that the current HDAC inhibitors (HDACis), mostly pan-HDACis, generally report disappointing results in clinical trials [13–18], specific inhibition of HDAC3 represents a novel therapeutic option for further clinical evaluation. It is therefore of high clinical value to maximize the anti-tumor effects of HDAC3 inhibition in the broader population of DLBCL patients, in addition to the subset of cases harboring *CREBBP* mutations.

In this study, we identify that knockout (KO) of *GNAS*, a gene encoding the G-protein α subunit (Gαs), sensitizes resistant lymphoma cells to HDAC3 inhibition using unbiased genome-wide CRISPR screening. We validate this sensitizing phenotype in multiple human and mouse lymphoma cell lines. Surprisingly, we show that *GNAS* KO-induced sensitization is independent of its canonical functions implicated in the Gαs–cyclic AMP (cAMP) signaling but rather caused by a novel mechanism of viral mimicry-related IFN responses, consistently associated with upregulation of double-stranded RNA (dsRNA) formation and transposable element (TE) expression. Importantly, *GNAS* KO-induced sensitization can be completely rescued by targeting the central IFN response modulator TBK1 or the key IFN receptor signaling mediator JAK1. In addition to the cell-intrinsic effects, we show that *GNAS* KO synergizes with HDAC3 inhibition to enhance CD8^+^ T cell-induced cytotoxicity. Furthermore, we observe that samples from DLBCL patients with low *GNAS* expression (“*GNAS* low”) display a high level of baseline TE expression and a pro-IFN state, and share common disrupted biological activities in histone modification, mRNA processing, and transcriptional regulation with *GNAS* KO DLBCL cells, suggesting a potential viral mimicry-primed condition in *GNAS* low DLBCL for enhanced response to HDAC3 inhibition. Overall, our findings unveil a previously unreported viral mimicry-related mechanism and could facilitate future clinical development of predictive and/or response biomarkers in the context of HDAC3 inhibition and lymphoma treatment.

## Results

### *GNAS* KO sensitizes resistant lymphoma cell lines to HDAC3 inhibition

**Fig. 1:**
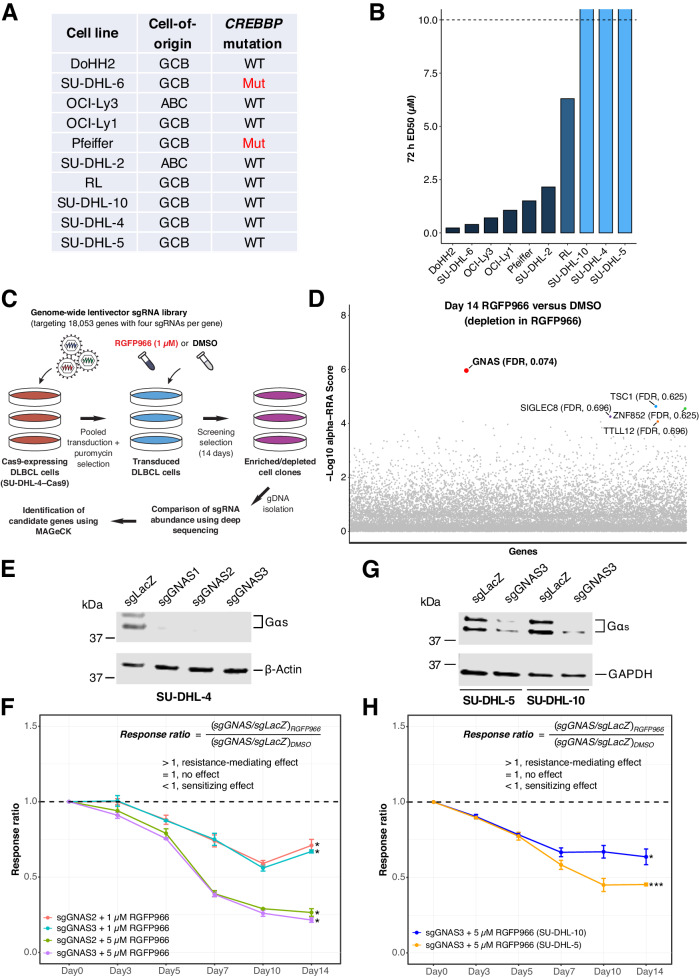
*GNAS* KO sensitizes resistant DLBCL cell lines to HDAC3 inhibition. **A** Ten DLBCL cell lines with different cell-of-origin and their *CREBBP* mutation statuses. SU-DHL-5 was detected to have a synonymous mutation and was classified as wild-type. **B** Response of 10 DLBCL cell lines to HDAC3 inhibitor RGFP966. Resistance is defined by 72 h ED50 > 10 µM (dotted line). **C** Schematic workflow of the genome-wide CRISPR KO screen for identifying sensitizers to RGFP966 in SU-DHL-4 cells. The Toronto KnockOut Library v3 (TKOv3) was used. **D** The top five sensitizing candidates ranked by modified robust rank aggregation (α-RRA) score with false discovery rate (FDR) shown. **E** Immunoblotting analysis of Gαs expression in SU-DHL-4 cells upon CRISPR–Cas9 gene editing. **F** *GNAS* KO sensitized SU-DHL-4 cells to RGFP966. **G** Immunoblotting analysis of Gαs expression in SU-DHL-5 and SU-DHL-10 cells upon CRISPR–Cas9 gene editing. **H** *GNAS* KO sensitized SU-DHL-5 and SU-DHL-10 cells to RGFP966. Response ratio represents the net effect of *GNAS* KO on cell response to RGFP966, considering the potential individual effects of sgGNAS, sgLacZ, and DMSO, calculated as shown. When the response ratio is less than one (dotted lines), the net effect is sensitization. Error bars represent ± SEM in **F** and **H** (*n* = 2 independent experiments). *, *p* < 0.05; ***, *p* < 0.001 (log2 transformed ratios on day 14 versus Log2(1) in both **F**, **H**; one-tailed unpaired Welch’s *t*-test). ABC, activated B-cell-like; GCB, germinal center B-cell-like; Mut, mutant; WT, wild-type.

To identify resistant models, we evaluated 10 DLBCL cell lines with different cell-of-origin and *CREBBP* mutation statuses for their response to RGFP966 [19], a highly selective HDAC3 inhibitor (Fig. 1A, B; see Supplementary Data 1 for details of *CREBBP* mutation analysis). While *CREBBP*-mutant cell lines were sensitive to RGFP966, three resistant cell lines with wild-type *CREBBP* (SU-DHL-4, SU-DHL-5, and SU-DHL-10) were identified with a median effective dose (ED50) > 10 µM at 72 h of treatment (Fig. 1B). To unbiasedly uncover sensitizers, we performed a genome-wide CRISPR KO screen in SU-DHL-4 cells treated with low-dose RGFP966 (1 µM) or the vehicle DMSO for 14 days (Fig. 1C). By analyzing the sequencing results for differential single guide RNA (sgRNA) representation using the statistical package Model-based Analysis of Genome-wide CRISPR/Cas9 Knockout (MAGeCK) [20], we obtained lists of ranked genes with a modified robust rank aggregation (α-RRA) score, *P*-value and false discovery rate (FDR) for each gene. Notably, the α-RRA scores are calculated based on sgRNA ranking and corrected for multiple hypothesis testing [20]. A gene with a small α-RRA score is likely a strong candidate because the sgRNAs targeting this gene are consistently ranked high. We first compared DMSO-treated samples collected on day 14 with those collected on day 0 to identify essential genes. Of all 800 essential genes (FDR < 0.1), *BCL2* was listed among the top candidates (α-RRA score = 1.38 × 10^−5^, *P*-value = 5.57 × 10^−5^, FDR = 0.008; Supplementary Data 2), in line with our previous genome-wide CRISPR screen performed in the same cell line [21], confirming the overall robustness of our screening platform for identifying sgRNA dropout. Next, we compared RGFP966-treated with DMSO-treated samples, both collected on day 14, to identify sensitizing candidates to HDAC3 inhibition. Among the top five sensitizing candidates ranked by α-RRA scores, *GNAS* was the top and only one identified with an FDR < 0.1 (α-RRA score = 1.18 × 10^−6^, *P*-value = 4.11 × 10^−6^, FDR = 0.074) while it was itself not an essential gene (Fig. 1D; see Supplementary Data 2 for full MAGeCK results of the ranked genes).

We next sought to validate the sensitizing effects of *GNAS* KO using individual SU-DHL-4 KO cell lines created by CRISPR–Cas9. All three *GNAS*-targeting sgRNAs, namely, sgGNAS1, 2, 3, resulted in successful gene KO confirmed by immunoblotting analysis of protein expression (Fig. 1E). In an initial test of 3-day treatment, sgGNAS2 and sgGNAS3 conferred cell sensitivity to RGFP966, with sgGNAS3 showing stronger effects, while sgGNAS1 had a trend toward sensitization (Supplementary Fig. 1A). We therefore further evaluated sgGNAS2 and sgGNAS3 for longer treatment durations up to 14 days. Both sgRNAs sensitized SU-DHL-4 cells to RGFP966 in a time- and dose-dependent manner (Fig. 1F). We repeated this validation process using sgGNAS3, the most effective sgRNA, in two additional resistant cell lines SU-DHL-5 and SU-DHL-10 and observed the sensitizing phenotype in both cell lines (Fig. 1G, H). Consistent with the screening results, strong sensitizing effects on survival and/or proliferation were only detected in *GNAS* KO cells treated with RGFP966, but not in control cells (Supplementary Fig. 1B), indicating a phenotype of synthetic lethality.

To further validate the specificity of our findings, we evaluated the effects of *GNAS* KO on another selective HDAC3 inhibitor BRD3308 [11], and an HDAC3-specific degrader XZ9002 [22]. These results recapitulated *GNAS* KO-induced sensitization to RGFP966 (Supplementary Fig. 1C–E). In contrast, *GNAS* KO did not result in differential sensitivity to the pan-HDACi romidepsin across a range of tested doses encompassing the full response spectrum (Supplementary Fig. 2), indicating that *GNAS* KO-induced sensitization appears specific to HDAC3 inhibition. We additionally examined *Gnas* KO in A20, a mouse lymphoma cell line showing moderate resistance to RGFP966 (72 h ED50 = 4.13 µM; Supplementary Fig. 1F) using individual KO cell lines (Supplementary Fig. 1G). In concordance with the human DLBCL cell lines, sensitization was observed when A20 *Gnas* KO cells were treated with RGFP966, with sgGNAS1 showing stronger effects (Supplementary Fig. 1H). Taken together, we identified and validated *GNAS* KO as a sensitizer to HDAC3 inhibition based on results of distinct sgRNAs and multiple HDAC3-targeting compounds in both human and mouse lymphoma cell lines, showing a consistent and conserved phenotype.

### *GNAS* KO-induced sensitization is independent of the Gαs–cAMP signaling axis

**Fig. 2:**
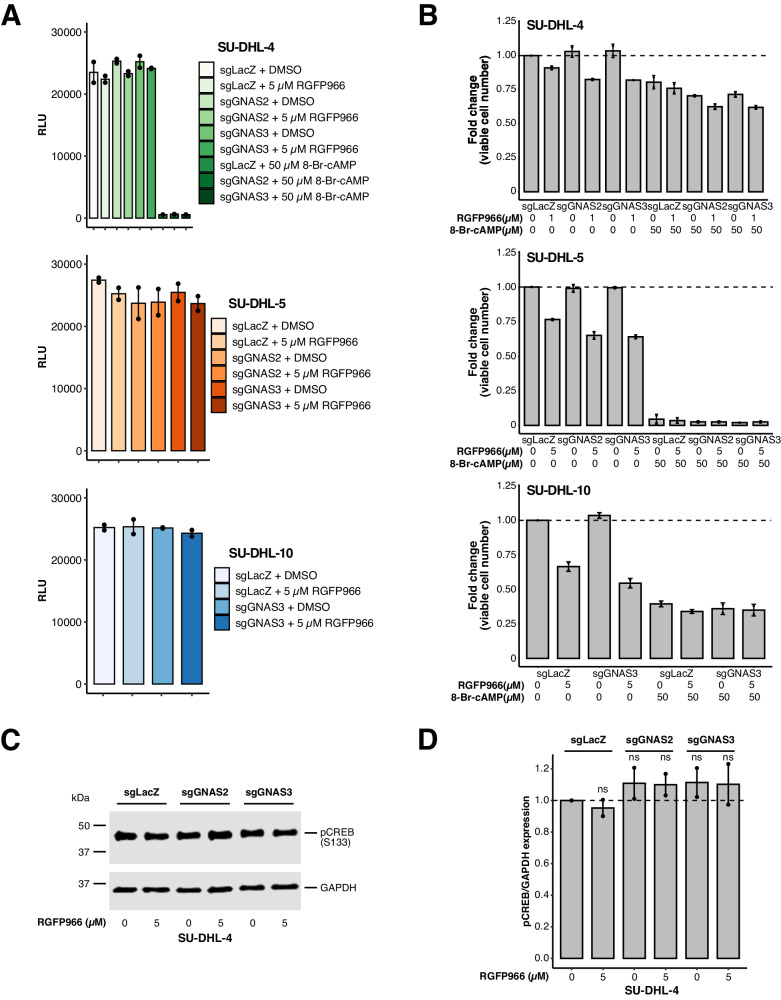
*GNAS* KO-induced sensitization is independent of the Gαs–cAMP signaling axis. **A** Detection of released cAMP levels under various conditions in DLBCL cell lines using a luminescence assay. Luminescence read in relative light units (RLU) is inversely proportional with cAMP levels as indicated by the addition of cAMP analog 8-Br-cAMP, used as a positive control. **B** Effects of 8-Br-cAMP on cell survival and/or proliferation in DLBCL cell lines. **C** Immunoblotting analysis of CREB phosphorylation at S133 in SU-DHL-4 cells. **D** Quantification of data in **C**. Cells were treated for five days before being harvested for downstream analysis in **A**–**D**. Error bars represent ± SEM in **A**, **B**, and **D** (*n* = 2 independent experiments). ns, not significant (log2 transformed ratios versus LacZ+DMSO in D; one-way ANOVA with Tukey’s HSD test for multiple comparisons).

The canonical function of Gαs is direct activation of adenylyl cyclases to convert ATP to the second messenger cAMP when activated by upstream G-protein-coupled receptor signaling [23]. Specifically, cAMP signaling has been reported to exert anti-lymphoma effects and, to the best of our knowledge, *GNAS* mutations are not recurrent in DLBCL and follicular lymphoma [24, 25]. To confirm whether the canonical Gαs–cAMP signaling axis is involved in the sensitizing phenotype, we first monitored released cAMP levels using a luminescence assay based on a luciferase reaction of available ATP consumed by cAMP-activated protein kinase A. In this assay, high light production indicates a high level of unconsumed ATP as a result of inactive protein kinase A and a low level of cAMP. In all three resistant DLBCL cell lines (*GNAS* KO or control) treated with RGFP966 or DMSO, the relative light units (RLU) values were very high compared with those detected from the positive control samples containing the long-lasting cAMP analog 8-Br-cAMP (Fig. 2A). All RLU values in *GNAS* KO cells were comparable with each other as well as the control cells, regardless of RGFP966 treatment (Fig. 2A), indicating that *GNAS* KO and/or RGFP966 had no effects on cAMP levels. In addition, 8-Br-cAMP did not reverse sensitization but further suppressed cell survival and/or proliferation (Fig. 2B), in line with the observations in the literature regarding the anti-lymphoma activity of cAMP signaling [25]. Furthermore, the key cAMP signaling downstream effector CREB was not differentially phosphorylated at its serine 133 regulatory site (Fig. 2C, D), suggesting that the signaling pathway was not specifically activated. In summary, these results did not support a role for canonical G-protein signaling in the sensitizing phenotype.

Given that HDAC3 inhibition has been shown to exert anti-lymphoma effects through increasing H3K27ac and promoting apoptosis [11], we sought to examine whether *GNAS* KO-induced sensitization is associated with those biological events. In agreement with the prior literature [11, 12], we found that RGFP966 alone was able to potentiate H3K27ac and moderately induce apoptosis (Supplementary Fig. 3A–D). However, *GNAS* KO plus RGFP966 did not result in further significant augmentation (Supplementary Fig. 3A–D). Similarly, RGFP966 alone caused moderate induction of cell cycle arrest but *GNAS* KO plus RGFP966 did not result in additional significant enhancement (Supplementary Fig. 3E, F). Hence, the sensitizing phenotype was unlikely a result of those individual activities induced by HDAC3 inhibition.

### *GNAS* KO-induced sensitization is dependent on IFN responses

**Fig. 3:**
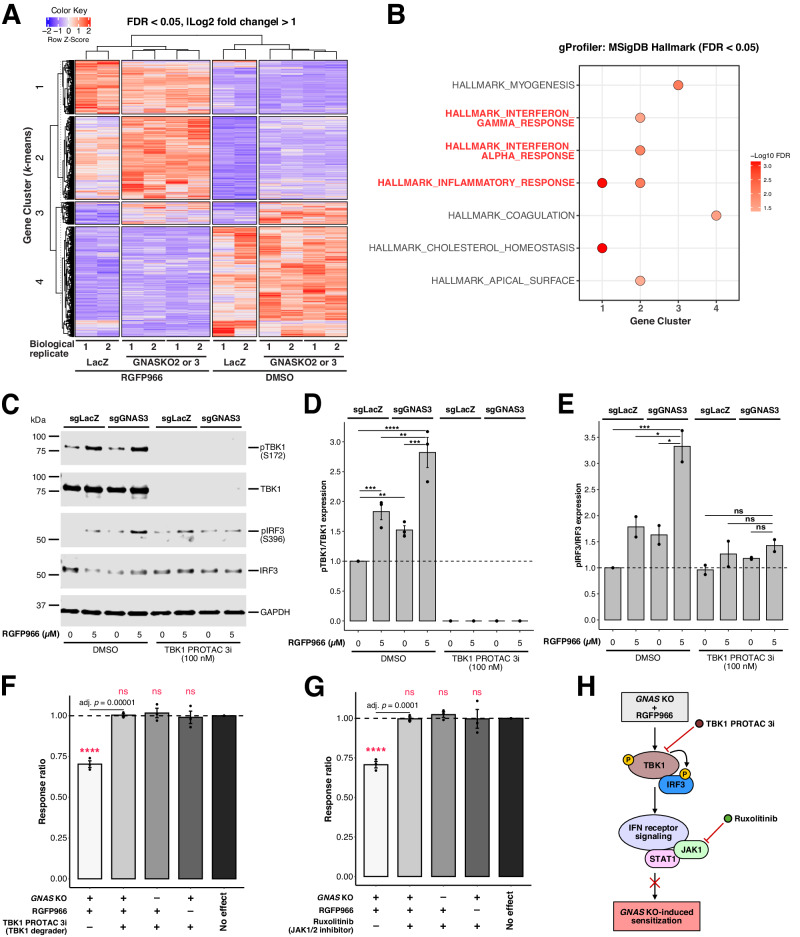
*GNAS* KO-induced sensitization to HDAC3 inhibition is dependent on IFN responses. **A** Heat map showing normalized expression dynamics of differentially expressed genes (FDR < 0.05 and |Log2 fold change | > 1) identified from cell-line bulk RNA-seq analysis. *K*-means clustering was applied, and four gene clusters were identified. **B** Pathway enrichment analysis of the genes identified from four clusters using the Molecular Signature Database (MSigDB) H collection by g:Profiler. Significantly enriched gene sets (FDR < 0.05) are highlighted by –Log10 FDR and the IFN response gene sets are highlighted in red. **C** Immunoblotting analysis of TBK1 phosphorylation at S172 and IRF3 phosphorylation at S396. **D** Quantification of TBK1 phosphorylation. **E** Quantification of IRF3 phosphorylation. **F**, **G** TBK1 degrader TBK1 PROTAC 3i at 100 nM **F** or JAK1/2 inhibitor ruxolitinib at 1 µM **G** rescued *GNAS* KO-induced sensitization to HDAC3 inhibition (RGFP966, 5 µM) without interacting with either *GNAS* KO or RGFP966. Cells were treated for five days before being harvested for downstream analysis in **A**–**G**. Error bars represent ± SEM in **D**–**G** (*n* = 3 independent experiments except for immunoblotting experiments detecting IRF3 phosphorylation where *n* = 2). *, *p* < 0.05; **, *p* < 0.01; ***, *p* < 0.001; ****, *p* < 0.0001; ns, not significant (ratios were log2 transformed for comparison in **D**–**G**; comparisons between conditions versus no effect were highlighted in red in **F**, **G**; one-way ANOVA with Tukey’s HSD test for multiple comparisons). **H** Schematic model of *GNAS* KO-induced sensitization via IFN receptor signaling. TBK1 is differentially phosphorylated at its S172 activation site upon *GNAS* KO plus RGFP966. IRF3 is then phosphorylated by TBK1 at its S396 activation site which activates IFN receptor signaling to induce sensitization. TBK1 PROTAC 3i and ruxolitinib rescue *GNAS* KO-induced sensitization by targeting upstream (TBK1) and downstream (JAK1) of IFN receptor signaling, respectively. P phosphorylation.

We showed above that *GNAS* KO-induced sensitization is independent of cAMP signaling, suggesting that novel mechanisms may underlie the sensitizing phenotype. To further explore the underlying mechanisms, we performed RNA sequencing (RNA-seq) to gain insights into the transcriptional profiles associated with sensitization. In total, we detected 361, 28, and 510 upregulated, and 226, 12, and 223 downregulated genes in RGFP966 alone, *GNAS* KO (sgGNAS3) alone, and *GNAS* KO (sgGNAS3) plus RGFP966 compared with the control condition, respectively (see Supplementary Data 3 for the full list of differentially expressed genes). To better understand gene signatures across the different conditions, we applied hierarchical clustering and identified four distinct gene clusters (Fig. 3A; see Supplementary Data 3 for the full gene list and clusters). Notably, Cluster 2 represents a unique group of upregulated genes in the combined *GNAS* KO plus RGFP966 condition while Cluster 1 contains genes upregulated mainly by RGFP966 and Cluster 3 represents genes upregulated in *GNAS* KO (Fig. 3A). Pathway enrichment analysis of all four gene clusters indicates that the significantly enriched gene sets were related to IFN responses, specifically in Cluster 2 but not in the other clusters (Fig. 3B), suggesting an activated IFN signature in the combined *GNAS* KO plus RGFP966 condition. Consistently, the IFN response gene sets were shown to be significantly upregulated by *GNAS* KO plus RGFP966 under pair-wise comparisons using Gene Set Enrichment Analysis (GSEA) (Supplementary Fig. 4A, B; see Supplementary Data 4 for the full results of GSEA analysis). To gain further insights into gene regulation, we subsequently performed an integrated analysis of assay for transposase-accessible chromatin with sequencing (ATAC-seq) and RNA-seq. Specifically, through our results from the motif enrichment analysis on the ATAC-seq data and functional enrichment analysis on the RNA-seq data, we identified two highly related members of the ETS family of transcription factors—PU.1 (encoded by *SPI1*) and SpiB (encoded by *SPIB*)—which were uniquely enriched in both analyses for *GNAS* KO plus RGFP966 (Supplementary Fig. 5). Interestingly, both PU.1 and SpiB have established roles in regulating IFN signaling and B-cell development [26, 27], indicating a molecular basis on transcriptional regulation of IFN signal activation by *GNAS* KO plus RGFP966.

We next investigated the relationship between *GNAS* KO-induced sensitization and IFN responses. Since activation of TBK1 and its effector IRF3 plays a central role in modulating IFN responses upon various upstream innate immune receptor signaling [28, 29], we sought to confirm whether TBK1 and IRF3 phosphorylation is increased by *GNAS* KO and/or HDAC3 inhibition. Indeed, immunoblotting analysis showed significant upregulation of TBK1 and IRF3 phosphorylation at their activation sites in *GNAS* KO plus RGFP966 compared with the other conditions (Fig. 3C–E), indicating a correlative relationship between *GNAS* KO-induced sensitization and activated IFN signaling. We then employed TBK1 PROTAC 3i [30], a highly selective TBK1 degrader, to functionally evaluate the relationship between the sensitizing phenotype and IFN signaling. At a low dose of 100 nM, TBK1 PROTAC 3i effectively degraded TBK1 protein to an undetectable level by both phospho- and total TBK1 antibodies (Fig. 3C) and abolished differential IRF3 phosphorylation in *GNAS* KO plus RGFP966 (Fig. 3E). Importantly, a complete reversal of sensitization was observed when TBK1 PROTAC 3i was added to *GNAS* KO cells treated with RGFP966 while it was shown to have no interaction with individual *GNAS* KO or RGFP966 (Fig. 3F), suggesting a causal relationship between sensitization and IFN responses.

In addition, we repeated the rescue experiment using ruxolitinib, a selective JAK1/2 inhibitor, to validate the results by targeting a key downstream mediator of IFN receptor signaling. Consistently, ruxolitinib phenocopied the complete rescue while showing no interaction with individual *GNAS* KO or RGFP966 (Fig. 3G). Collectively, we confirmed that *GNAS* KO-induced sensitization to HDAC3 inhibition was mediated through IFN responses using a functional validation approach involving both upstream and downstream regulators of IFN receptor signaling (Fig. 3H).

### *GNAS* KO synergizes with HDAC3 inhibition to enhance CD8^+^ T cell-induced cytotoxicity

**Fig. 4:**
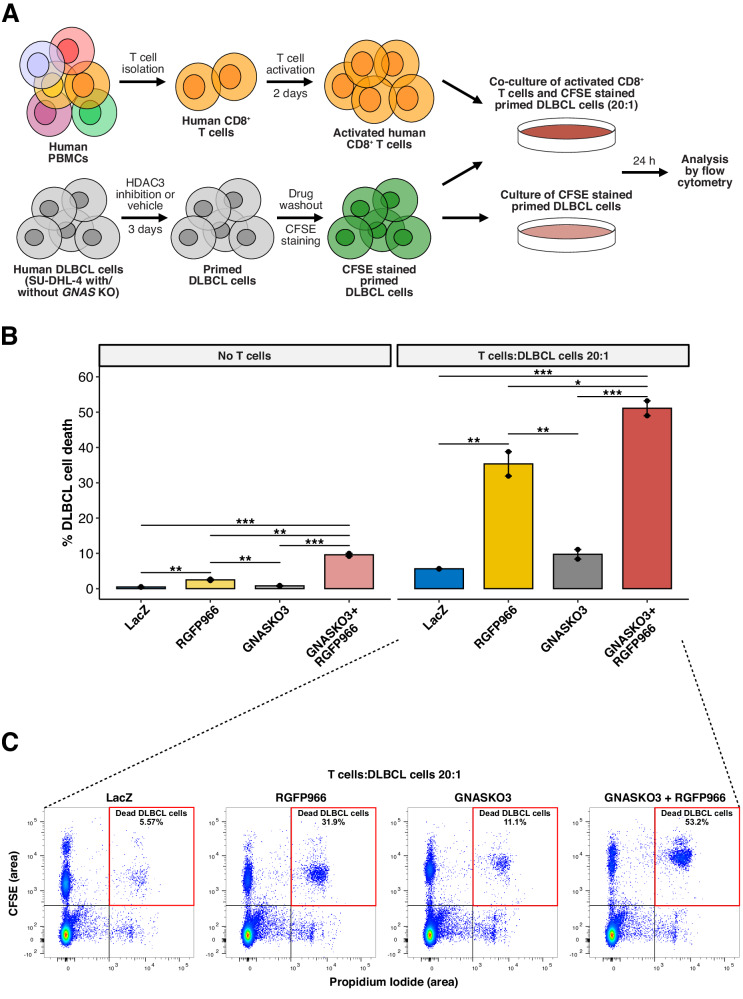
*GNAS* KO synergizes with HDAC3 inhibition to enhance CD8^+^ T cell-induced cytotoxicity. **A** Schematic representation of the T cell co-culture cytotoxicity assay. **B** Quantification of percentage cell death in DLBCL cells. Error bars represent ± SEM (*n* = 2 independent experiments). *, *p* < 0.05; **, *p* < 0.01; ***, *p* < 0.001 (log2 transformed values or values were compared; one-way ANOVA with Tukey’s HSD test for multiple comparisons). **C** Flow cytometry-based determination of cell death. Single cells and non-debris were included for analysis of 5-(and 6)-Carboxyfluorescein diacetate succinimidyl ester (CFSE) and propidium iodide (PI) signals. CFSE positive and negative populations represent DLBCL cells and CD8^+^ T cells, respectively. PI positive and negative groups represent dead and live cells, respectively. The total number of single DLBCL cells acquired per sample was 10,000. The percentages shown are percentage cell death within the total DLBCL cell population per sample. PBMCs, peripheral blood mononuclear cells.

Given the activated IFN signature observed in the combined *GNAS* KO plus RGFP966 condition and the critical role of IFN signaling in anti-tumor immune responses, we explored whether *GNAS* KO can synergize with HDAC3 inhibition to promote anti-tumor immunity such as CD8^+^ T cell-induced cytotoxicity. We therefore performed a T cell co-culture cytotoxicity assay by directly co-culturing anti-CD3/CD28-activated human CD8^+^ T cells with RGFP966 pre-treated SU-DHL-4 cells (Fig. 4A). Without T cells, RGFP966 alone induced moderate cell death (~3%) while *GNAS* KO plus RGFP966 promoted cell death to ~10% (Fig. 4B). Strikingly, 24 h co-culture of activated CD8^+^ T cells and SU-DHL-4 cells resulted in enhanced DLBCL cell death under all conditions, and such effects were particularly prominent when SU-DHL-4 cells were pre-treated with RGFP966 (Fig. 4B, C). Moreover, CD8^+^ T cell-induced cytotoxicity was significantly promoted by *GNAS* KO plus RGFP966 (~51%) versus RGFP966 alone (~35%) (Fig. 4B, C). In summary, our results showed that *GNAS* KO not only sensitized DLBCL cells to HDAC3 inhibition through cell-intrinsic IFN responses but also potentiated CD8^+^ T cell-induced cytotoxicity with HDAC3 inhibition.

### *GNAS* KO plus HDAC3 inhibition is associated with dsRNA formation and TE expression for viral mimicry induction

**Fig. 5:**
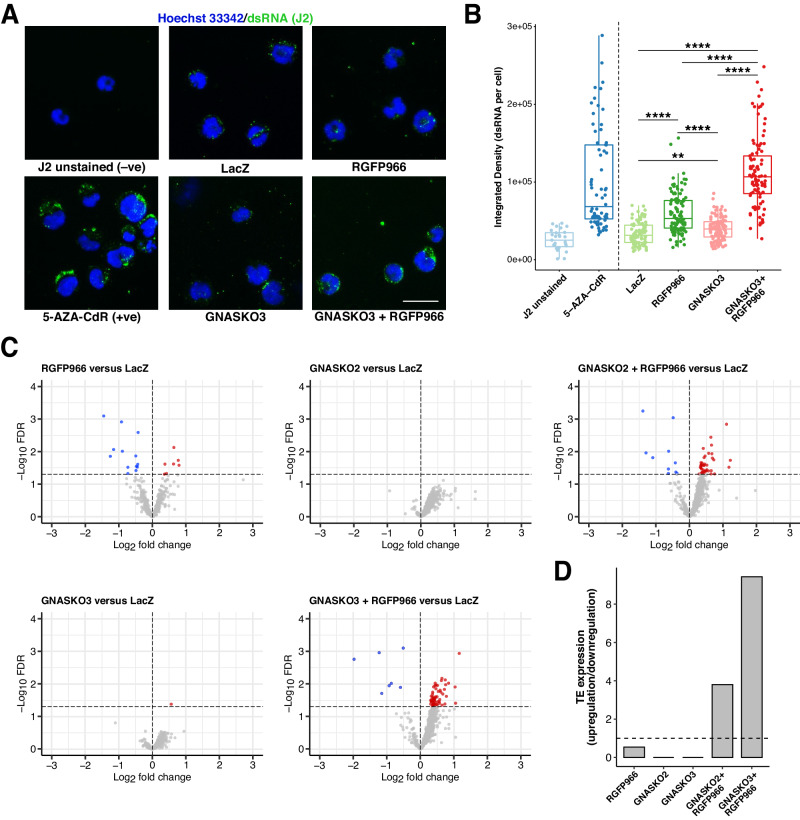
*GNAS* KO plus RGFP966 is associated with dsRNA formation and TE expression for viral mimicry induction. **A** Representative images of SU-DHL-4 cells stained by Hoechst 33342 (for cell nuclei; blue) and J2 antibody (for dsRNAs; green) from immunofluorescence analysis. Control LacZ cells omitting primary J2 antibody and treated with 300 nM 5-AZA-CdR were the negative and positive control, respectively. Scale bar, 25 µm. **B** Quantification of results in **A**. **p < 0.01; ****p < 0.0001 (Kruskal–Wallis test with Wilcoxon rank sum test for pairwise comparisons). **C** Volcano plots showing normalized expression dynamics of differentially expressed TEs (FDR < 0.05 and |Log2 fold change | > 0) identified from TEtranscripts analysis of cell-line bulk RNA-seq data. Upregulated and downregulated TEs are highlighted in red and blue, respectively. The horizontal and vertical dotted lines indicate FDR = 0.05 and Log2 fold change = 0, respectively. **D** Normalized TE expression under each comparison by a ratio of upregulated versus downregulated TEs shown in **C**. The dotted line represents normalized TE expression = 1 (equal numbers of upregulated and downregulated TEs).

Viral mimicry is a cellular state of activated antiviral responses triggered by endogenous viral infection-irrelevant stimuli [29]. Recent studies have reported the induction of viral mimicry and IFN responses after treatment with epigenetic agents in various cancer models [31, 32]. More specifically, DNA methyltransferase inhibitors (DNMTis) such as 5-azacytidine (AZA) and 5-Aza-2′-deoxycytidine (5-AZA-CdR) are shown to derepress transcription of repetitive TE sequences, particularly retrotransposons such as endogenous retroviruses from the long-terminal repeat (LTR) family, leading to the formation of dsRNAs and induction of robust cell-intrinsic anti-tumor IFN responses [31, 32]. To confirm whether viral mimicry is involved, we first evaluated whether the formation of cytoplasmic dsRNAs is evident in *GNAS* KO plus RGFP966. Using immunofluorescence with the J2 antibody, a gold standard for detecting dsRNAs [33], we validated our experimental conditions using 5-AZA-CdR [32], and demonstrated a robust increase of cytoplasmic dsRNA formation in *GNAS* KO plus RGFP966 while such increase was significant yet moderate in the individual condition of *GNAS* KO or RGFP966 in SU-DHL-4 cells (Fig. 5A, B). Additionally, consistent with the sensitizing phenotype (Supplementary Fig. 1H), cytoplasmic dsRNA formation was also significantly upregulated by *Gnas* KO plus RGFP966 in the mouse lymphoma cell line A20 (Supplementary Fig. 6A, B).

We next explored TE expression from cell-line bulk RNA-seq data using TEtranscripts [34], one of the most widely used tools for TE transcriptome analysis. The TE family-specific quantification from TEtranscripts analysis confirmed a profound increase of TE expression in *GNAS* KO plus RGFP966, not only reflected from the absolute number of upregulated TEs but also the relative ratio where the number of upregulated TEs was normalized by that of downregulated TEs (Fig. 5C, D). In contrast, RGFP966 alone showed mildly upregulated TEs with a higher level of downregulation, and *GNAS* KO alone resulted in minimal changes in TE expression (Fig. 5C, D). Nevertheless, the overall trend in *GNAS* KO alone appeared to be upregulation as most TEs had a positive Log2 fold change (Fig. 5C), suggesting a potential role of *GNAS* KO in inducing viral mimicry with HDAC3 inhibition. Furthermore, with a focus on the upregulated TEs, we identified distinct TE subfamilies from TEtranscripts analysis including those belonging to retrotransposons—short interspersed nuclear elements (SINEs), long interspersed nuclear elements (LINEs), and LTR elements—and DNA transposons (Supplementary Fig. 6C), all seemed to be strongly upregulated in *GNAS* KO plus RGFP966 compared with the individual conditions. We finally investigated whether TE upregulation is correlated with epigenetic regulation. By performing ATAC-seq, we detected a high number of upregulated differential accessibility sites (DASs) at TEs in *GNAS* KO plus RGFP966 while only a few DASs were observed in RGFP966 alone and none in *GNAS* KO alone (Supplementary Fig. 6D), revealing a similar pattern when compared with the TE transcriptome analysis (Fig. 5C, D; Supplementary Fig. 6C). The TEs identified from ATAC-seq analysis also included those belonging to SINEs, LINEs, LTR elements, and DNA transposons, on a locus-specific basis (Supplementary Fig. 6D). Our results suggest that the increased TE expression in *GNAS* KO plus RGFP966 was, at least, in part attributed to increased chromatin accessibility and potential reactivation of TE transcription. Taken together, we demonstrated a prominent elevation of dsRNA formation, TE expression, and chromatin accessibility in *GNAS* KO plus RGFP966 while such events were not strongly evident in either individual condition, indicating a state of viral mimicry induction for activating IFN responses to mediate sensitization.

### Characterization of *GNAS* expression on TE expression and pathway enrichment in DLBCL patients

**Fig. 6:**
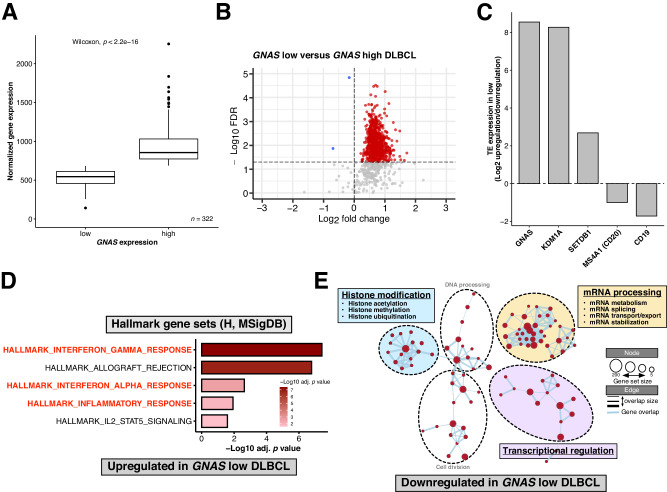
Characterization of *GNAS* expression on TE expression and pathway enrichment in DLBCL patients. **A** Classification of *GNAS* low and high groups based on median *GNAS* expression in DLBCL patients. DLBCL samples were collected at diagnosis (Ennishi et al. 2019; *n* = 322). **B** Volcano plot showing normalized expression dynamics of differentially expressed TEs (FDR < 0.05 and |Log2 fold change | > 0) identified from TEtranscripts analysis of DLBCL patient bulk RNA-seq data. Upregulated and downregulated TEs are highlighted in red and blue, respectively. The horizontal and vertical dotted lines indicate FDR = 0.05 and Log2 fold change = 0, respectively. **C** Normalized TE expression under each comparison by a Log2 ratio of upregulated versus downregulated TEs. The dotted line represents normalized TE expression = Log2(1) (equal numbers of upregulated and downregulated TEs). All comparisons shown in **B**, **C** were made between low versus high expression. **D** Pathway enrichment analysis of the genes upregulated (FDR < 0.05 and Log2 fold change > 0.5) in *GNAS* low DLBCL patients using the MSigDB H collection by g:Profiler. Significantly enriched gene sets (FDR < 0.05) are highlighted by –Log10 FDR and the IFN response gene sets are highlighted in red. **E** Cytoscape-generated enrichment maps showing gene network clusters based on GSEA using the MSigDB C5 GO subcollection (FDR < 0.3). GSEA was performed for the comparison between *GNAS* high versus low. Each node represents a gene set and links represent gene overlap between sets. Three major biological themes histone modification, mRNA processing, and transcriptional regulation are identified and highlighted.

Evidence has shown that certain conditions, characterized by high baseline retrotransposon expression and dsRNA levels with optional IFN activation, can prime cancers for viral mimicry induction and anti-tumor activity upon treatment with epigenetic therapy (“viral mimicry priming”) [29, 35]. Our TE transcriptome analysis and immunofluorescence results indicate that *GNAS* KO may play a role in priming resistant lymphoma to viral mimicry induction upon HDAC3 inhibition. Hence, we sought to characterize *GNAS* expression in DLBCL patient samples in the context of viral mimicry priming. From a cohort of 322 DLBCL patients [36], we first grouped the transcriptome samples according to the median *GNAS* expression (Fig. 6A). Using TEtranscripts, we identified differentially expressed TEs in *GNAS* low versus *GNAS* high DLBCL patients (Fig. 6B). Strikingly, the *GNAS* low group showed predominant TE upregulation over downregulation (Fig. 6C), consistent with the known epigenetic regulators such as the histone demethylase KDM1A (LSD1) [37, 38] and histone methyltransferase SETDB1 [39–41] whose loss or inhibition results in TE expression, dsRNA enrichment, and/or IFN induction (Fig. 6C; Supplementary Fig. 7A, B). To exclude the possibility that *GNAS* low-associated TE upregulation was due to stromal/immune cell enrichment, we additionally assessed TE expression for two B-cell-specific markers *MS4A1 (CD20)* and *CD19*. Both the *MS4A1* and *CD19* low groups displayed a higher level of TE downregulation over upregulation (Fig. 6C; Supplementary Fig. 7C, D), suggesting that *GNAS* low-associated TE upregulation was tumor-intrinsic. Interestingly, the distribution of upregulated TEs in the *GNAS* low group was consistent with those in the *KDM1A* and *SETDB1* low groups (Supplementary Fig. 8A), showing a high proportion of retrotransposons, especially LTR elements.

We further performed pathway enrichment analysis to gain insights into whether *GNAS* expression is correlated with altered biological activities in DLBCL. In line with TE upregulation, IFN response gene sets were shown to be significantly enriched in the *GNAS* low group (Fig. 6D). In addition, we used EnrichmentMap [42] to visualize networks of enriched gene sets which revealed that three major biological themes, i.e., histone modification, mRNA processing, and transcriptional regulation were downregulated in the *GNAS* low group (Fig. 6E; see Supplementary Data 5 for the full results of GSEA analysis). Markedly, these results were validated using an independent cohort of 481 DLBCL transcriptome samples [43] following the same analytical procedure (Supplementary Fig. 8B–D; see Supplementary Data 5 for the full results of GSEA analysis). Intriguingly, the *GNAS* low-associated downregulated terms identified from the two independent clinical DLBCL cohorts were also detected in the cultured *GNAS* KO human DLBCL cells (both sgGNAS2 and sgGNAS3; Supplementary Fig. 9; see Supplementary Data 5 for the full results of GSEA analysis), indicating common cell-intrinsic effects of *GNAS* deficiency on DLBCL biology. In summary, we demonstrated that low *GNAS* expression was associated with high baseline tumor-intrinsic TE expression and a pro-IFN state in DLBCL patients, suggesting its potential role in viral mimicry priming. In addition, we showed common biological activities affected by low *GNAS* expression in DLBCL patients and *GNAS* KO in DLBCL cells, shedding light on the putative molecular mechanisms underlying viral mimicry priming.

## Discussion

In this study, we report *GNAS* KO as a molecular sensitizer to HDAC3 inhibition in resistant lymphoma cells. Mechanistically, we show that the sensitizing phenotype is independent of the canonical Gαs–cAMP signaling axis but caused by viral mimicry-related IFN responses. *GNAS* KO further synergizes with HDAC3 inhibition to enhance CD8^+^ T cell-induced cytotoxicity. By exploring two independent clinical cohorts of DLBCL transcriptomic data, we reveal that low *GNAS* expression is associated with a high level of baseline TE expression and upregulated IFN signaling, and shares common disrupted biological activities in histone modification, mRNA processing, and transcriptional regulation with *GNAS* KO DLBCL cells.

Isoform-selective inhibition of HDACs offers unique advantages over traditional pan-HDACis by avoiding targeting multiple metalloenzymes thus exhibiting more favorable toxicity profiles [44]. Based on a strong rationale that B-cell lymphomas with *CREBBP* mutations are dependent on HDAC3 activity, HDAC3 inhibition has been recently demonstrated as a promising strategy for those mutant cases while wild-type tumors show modest responses [6, 11]. In line with this observation, the three HDAC3 inhibition-resistant DLBCL cell lines we identified from the initial screen of cell response were all detected as wild-type according to our mutation analysis of targeted DNA sequencing. In our CRISPR screen, we detected KO of *GNAS* as the top sensitizing candidate from a library containing more than 72,000 sgRNAs targeting more than 18,000 genes, indicating a high specificity of our results. Our following validation using alternative HDAC3-specific targeting compounds, a pan-HDACi, and multiple sgRNAs as well as both human and mouse lymphoma cell lines further confirmed the on-target effects and general applicability. Hence, our models provided a solid platform to study the mechanisms underlying *GNAS* KO-induced sensitization to HDAC3 inhibition.

*GNAS* is frequently mutated in many solid tumors where the hotspot R201 activating mutations often result in constitutive activation of Gαs–cAMP signaling, creating an oncogenic addiction [45–49]. On the contrary, *GNAS* has been identified as a potent tumor suppressor in sonic Hedgehog-driven medulloblastoma which displays a substantial loss of *GNAS*-containing chromosomal region [50]. In lymphoma, however, *GNAS* mutations are rare (0.21% in DLBCL and 0% in follicular lymphoma) with no *GNAS* activating mutations reported [24]. Furthermore, cAMP signaling has been shown to exert anti-lymphoma effects by suppressing growth and inducing apoptosis [25]. These results suggest that GNAS has organ- and tumor-specific functions and its baseline activity of activating adenylyl cyclases in lymphoma may be minimal due to cAMP-induced anti-lymphoma effects. Consistently, we demonstrated that the addition of cAMP analog 8-Br-cAMP led to a varied degree of anti-tumor effects in all three DLBCL cell lines and *GNAS* KO did not alter cAMP levels which appeared to be already low in the control cells. Combining the observations that *GNAS* KO did not alter cAMP downstream signaling and had no significant effects on cell survival and/or proliferation, apoptosis, and cell cycle progression, we propose that GNAS has novel biological functions in lymphoma.

In *CREBBP*-mutant B-cell lymphoma cells, HDAC3 inhibition shows strong anti-tumor effects via H3K27ac restoration [11]. In HDAC3 inhibition-resistant *CREBBP* wild-type cells, we observed elevated H3K27ac, moderate induction of apoptosis, and mild cell cycle arrest by RGFP966 alone. These HDAC3 inhibition-directed changes were unlikely being sufficient to explain *GNAS* KO-induced sensitization because none of those were strongly potentiated in *GNAS* KO plus RGFP966. Instead, we showed a novel mechanism of viral mimicry-related IFN responses underlying the sensitizing phenotype. In agreement with the previous findings that pan-HDACis alone induced LTR transcription but did not stimulate AZA-induced immune and viral defense genes [51], our results demonstrated that HDAC3 inhibition alone did not trigger obvious IFN responses while showing a moderate level of increased TE expression and dsRNA formation. With *GNAS* KO, those events were significantly enhanced. In particular, epigenetic therapy-induced derepression of retrotransposon transcription results in the formation of cytoplasmic repetitive transcript-derived dsRNAs [29, 31, 32, 35]. Upon dsRNAs binding to cytosolic or endosomal RNA sensors, MAVS is activated to initiate a phosphorylation cascade involving TBK1 and IRF3, eventually leading to activated IFN receptor signaling through JAK and STAT [28, 29, 31, 32]. In addition to retrotransposon-derived dsRNA-sensing, the dsDNA-sensing pathway stimulates STING and phosphorylation of TBK1 and IRF3, also resulting in viral mimicry activation [28, 29]. Together with our results showing upregulated dsRNA formation and TE expression, our functional evaluation based on TBK1, IRF3, and JAK1 provides direct evidence for the causal link between *GNAS* KO-induced sensitization and viral mimicry induction regardless of the upstream inputs, addressing a general knowledge gap between isoform-selective HDAC inhibition (e.g., HDAC3 inhibition) and viral mimicry in cancer. Although we further showed upregulated retrotransposon expression and increased chromatin accessibility at retrotransposons from RNA-seq and ATAC-seq analysis, respectively, suggesting a role of retrotransposon derepression, further investigation is warranted to understand the exact molecular upstream mechanisms of viral mimicry induction. In addition to retrotransposon alterations, our integrated analysis of ATAC-seq and RNA-seq further reveals a potential link between *GNAS* KO plus HDAC3 inhibition and the ETS family transcription factors PU.1 and SpiB for transcriptional regulation of IFN signal activation in B-cell lymphoma. More specifically, PU.1 has been reported to be downregulated in primary effusion lymphoma, and its restoration results in the induction of pro-apoptotic IFN-stimulated genes [26], providing supporting evidence for our findings. Given these results, future studies are required to understand the broader implications of the relationship between *GNAS* KO, HDAC3 inhibition, and ETS family transcription factors in regulating IFN responses.

HDAC3 inhibition, viral mimicry and cell-intrinsic IFN responses have been associated with enhanced anti-tumor immunity and synergy with immunotherapy such as checkpoint inhibitors [11, 12, 29, 52]. In line with those findings, we showed that HDAC3 inhibition strongly potentiated CD8^+^ T cell-induced cytotoxicity and *GNAS* KO further promoted such cytotoxic effect. This enhanced cytotoxicity did not appear to be due to MHC–T cell receptor signaling, as the CD8^+^ T cells were not primed with the target DLBCL cells. Given that activated CD8^+^ T cells secrete a range of pro-inflammatory cytokines [53], it is likely that the enhanced cytotoxicity is related to the further stimulation of intrinsic IFN responses by T cell-produced cytokines such as IFN-gamma [54]. While these results provide an interesting perspective on the potential immune responses associated with *GNAS* KO and HDAC3 inhibition, further studies are needed to confirm the effects of cytokines and explore the role of tumor neoantigens and upregulation of MHC complexes in enhancing T cell-induced cytotoxicity as well as the effects of immunotherapy such as checkpoint inhibitors and CAR-T cell therapy.

When cancers show high baseline TE expression and dsRNA formation, they are considered “primed” for viral mimicry induction because treatment with epigenetic therapy can trigger further TE expression and dsRNA formation beyond IFN tolerance thresholds [29, 35]. For example, in high-grade gliomas with *H3.3*^*K27M*^ mutations, increased retrotransposon expression due to loss of repressive H3K27 trimethylation mark renders tumors more sensitive to DNMTi and/or HDACi via enhanced IFN responses compared with wild-type tumors [55]. Although our cell-line data showed minor dsRNA formation with a trend of TE upregulation by *GNAS* KO, we found using a cohort of 322 DLBCL transcriptome samples that low *GNAS* expression was associated with strong tumor-intrinsic upregulation of retrotransposon expression. This is consistent with our analysis of the histone demethylase KDM1A (LSD1) [37, 38] and histone methyltransferase SETDB1 [39–41] whose loss or inhibition is associated with viral mimicry induction using the same dataset. Importantly, together with validation using an additional independent cohort of 481 DLBCL transcriptome samples as well as analysis of our cell-line data, we identified three common biological themes—histone modification, mRNA processing, and transcriptional regulation—that were disrupted in both *GNAS* low DLBCL patients and *GNAS* KO DLBCL cells. This commonality not only rationalizes a potential viral mimicry priming role of low *GNAS* expression in DLBCL patients but also opens an avenue to address the questions above regarding the potential novel functions of GNAS in lymphoma and how *GNAS* KO plus HDAC3 inhibition initiates viral mimicry induction. To illustrate, in addition to the processes of histone modification and transcriptional regulation which have been shown to modulate TE expression and dsRNA formation [29, 35, 56], altered mRNA processing such as mRNA modification (e.g., methylation) [56, 57], splicing [56, 58, 59], metabolism [56], and stability [52] are also implicated. Hence, future studies could focus on investigating the potential functions of GNAS in those biological activities.

In conclusion, we identify that *GNAS* KO is a sensitizer of resistant lymphoma cells to HDAC3 inhibition via viral mimicry-related IFN responses and can enhance CD8^+^ T cell-induced cytotoxicity with HDAC3 inhibition, and further demonstrate a potential viral mimicry priming role of low *GNAS* expression in DLBCL patients. Ultimately, our findings could facilitate the clinical development of predictive and/or response biomarkers for the rational use of HDAC3-targeting agents in lymphoma, especially the *CREBBP* wild-type cases.

## Methods

### Chemicals

The selective HDAC3 inhibitors RGFP966 and BRD3308 (Selleckchem), the pan-HDAC inhibitor romidepsin (Selleckchem), the cAMP analog 8-Br-cAMP (Tocris), the TBK1 degrader TBK1 PROTAC 3i (Tocris), the JAK1/2 inhibitor ruxolitinib (Selleckchem), and the DNA methyltransferase inhibitor 5-Aza-2’-deoxycytidine (5-AZA-CdR; MedChemExpress) were reconstituted and stored according to the manufacturer’s instructions. The HDAC3-specific degrader XZ9002 and its control compound XZ9002-NC were provided by Drs. Yufeng Xiao and Guangrong Zheng at the University of Florida.

### Cell lines

The human DLBCL cell lines SU-DHL-4 (RRID:CVCL_0539), SU-DHL-5 (RRID:CVCL_1735), and SU-DHL-10 (RRID:CVCL_1889) were cultured in RPMI 1640 medium supplemented with 20% FBS. The human DLBCL cell lines DoHH2 (RRID:CVCL_1179), SU-DHL-6 (RRID:CVCL_2206), OCI-Ly1 (RRID:CVCL_1879), Pfeiffer (RRID:CVCL_3326), SU-DHL-2 (RRID:CVCL_9550), and RL (RRID:CVCL_1660) were cultured in RPMI 1640 medium supplemented with 10% FBS. The human DLBCL cell line OCI-Ly3 (RRID:CVCL_8800) was cultured in Iscove’s Modified Dulbecco’s Medium (IMDM) supplemented with 20% FBS. The mouse lymphoma cell line A20 (RRID:CVCL_1940) was cultured in RPMI 1640 medium supplemented with 10% FBS and 0.05 mM 2-mercaptoethanol. The human embryonic kidney 293 T (HEK293T) cell line was cultured in Dulbecco’s Modified Eagle Medium (DMEM) supplemented with 10% FBS and treated with trypsin-EDTA during detachment. Genetically modified cell lines were generated as described using CRISPR–Cas9 (see more details in Supplementary Methods). All cell lines were incubated in 5% CO_2_ at 37 °C. All cell lines were authenticated using short tandem repeat profiling and verified free of mycoplasma contamination.

### Genome-wide CRISPR screen and analysis

Approximately 850 million Cas9-expressing SU-DHL-4 cells (SU-DHL-4–Cas9) were transduced with lentivectors encoding the Toronto KnockOut Library v3 (TKOv3) [60] at a multiplicity of infection of 0.3 and 400× library coverage. Positively transduced and sgRNA-integrated cells were selected using puromycin for three days. Puromycin-resistant cells were subsequently pooled and divided into two arms of triplicates, with 28.5 million cells per replicate (vehicle DMSO, i.e., control arm or 1 μM RGFP966, i.e., treatment arm). Cell numbers for each replicate were maintained at 28.5 million at each passage throughout the screen. On day 0 (T0), the reference set was frozen down immediately after puromycin selection. The control and treatment arms were further incubated for 14 days and 28.5 million cells per replicate were collected (T14). Genomic DNA (gDNA) was isolated using the QIAamp Blood Maxi Kit (QIAGEN). To generate the next-generation sequencing library, sgRNA-encoding cassettes were amplified from gDNA using a 2-step PCR protocol to attach the sequencing adapter and index sequences (Princess Margaret Genomics Centre). The purified amplicon products were quantified using an Illumina Library Quantification Kit (Kapa Biosystems) and sequenced on a NovaSeq 6000 system (Illumina). Normalization and alignment of the reads were performed using Model-based Analysis of Genome-wide CRISPR/Cas9 Knockout (MAGeCK; v0.5.7) [20]. Depletion of sgRNAs was analyzed by comparing the normalized reads and the candidate genes were determined based on the sgRNA ranking results with modified robust rank aggregation (α-RRA) scores and multiple comparisons.

### alamarBlue assay for cell viability

Cells were used to seed 96-well flat clear-bottom polystyrene microplates at 25,000 or 50,000 per well in culture media plus drugs at various concentrations or DMSO for 72 h. Resazurin (R7017, Sigma) solution (0.015% in PBS) was added to each well (10% of culture volume) and plates were incubated in 5% CO_2_ at 37 °C for 4 h. Fluorescence (560/590) was measured using the Spectramax M5 microplate reader (Molecular Devices). ED50 was calculated using the R package drfit (v0.7.2).

### Assay evaluating cell survival and/or proliferation

Cells were used to seed 24-well plates, 6-well plates, or T25 flasks at 100,000/mL or 25,000/mL in culture media plus drugs at the designated concentrations or DMSO for the designated duration. Cells were counted using a Countess II automated cell counter according to the manufacturer’s instructions. Response ratios were adapted from the small-molecule inhibitor assay [61] and calculated based on the normalized ratios of viable cell numbers under different conditions (see Fig. 1F, H for examples).

### Cell-line bulk RNA-seq and analysis

SU-DHL-4 cells were treated with RGFP966 (5 µM) or DMSO for five days before total RNA was isolated using RNeasy Mini Kit (QIAGEN) according to the manufacturer’s instructions. The samples were quantified by Qubit RNA Kit (Life Technologies) and quality was assessed by Agilent Bioanalyzer. All samples had RIN ≥ 9.4. Libraries were prepared with 100 ng of total RNA using Stranded Total RNA prep with Ribo-Zero Plus kit (Illumina) with 13 cycles of amplification used. Final cDNA libraries were size validated using Agilent Bioanalyzer or Tapestation and concentration validated by qPCR (Kapa Biosystems/Roche). All libraries were normalized and pooled together, denatured with 0.2 N NaOH, and diluted to a final concentration of 250 pM. Pooled libraries were loaded onto an Illumina Novaseq V1.5 cartridge for cluster generation and sequencing on a NovaSeq 6000 system (Illumina) Paired-end 101 bp protocol to achieve ~40 million reads per sample. The raw FASTQ files were first processed by trimming the low-quality reads and adapters using fastp (v0.23.2) [62]. The clean reads were then mapped to the reference genome (GRCh38, with GENCODE annotations from V38 (Ensembl 104)) using the STAR aligner (v2.7.9a) [63]. Aligned BAM files were subsequently collected as input to the counting tool HTSeq-count (v0.11.0) [64] to obtain the read count per gene per sample, then merged into a count matrix for differential analysis. Differential gene expression analysis was performed using edgeR (v3.32.1) [65]. Significance was defined by FDR < 0.05 and |Log2 fold-change | > 1. The heat map was generated using ComplexHeatmap (v2.12.1) [66] and the gene clusters were defined according to *k*-means.

### Pathway enrichment analysis

Pathway enrichment analysis was performed using g:Profiler (version: e110_eg57_p18_4b54a898) [67] with g:SCS multiple testing correction method applying significance threshold of 0.05 and with the Molecular Signatures Database (MSigDB; the H collection of hallmark gene sets, v2023.1) or using Gene Set Enrichment Analysis (GSEA; v4.3.2) [68] with the Molecular Signatures Database (MSigDB; the C5 GO subcollection of Gene Ontology gene sets, v2023.1). Enrichment maps were generated using Cytoscape (v3.10.0) [69] and its application EnrichmentMap (v3.3.5) [42]. For g:Profiler analysis, a custom background was applied by inserting all the expressed genes from the RNA-seq analysis.

### Detection and analysis of TE expression

The clean RNA-seq reads were mapped to the GRCh37 reference genome repository using the STAR aligner for TE analysis. The reference sequences and gene GTF file were downloaded from https://ftp.ebi.ac.uk/pub/databases/gencode/Gencode_human/release_37/. BAM files (aligned and sorted by coordinate) were used for quantification of TE expression using TEtranscripts (v2.2.3; https://github.com/mhammell-laboratory/TEtranscripts) [34]. The TEcount function was run with the default parameters using the curated gene GTF file and TE GTF file (GRCh37_GENCODE_rmsk_TE.gtf; downloaded from https://labshare.cshl.edu/shares/mhammelllab/www-data/TEtranscripts/TE_GTF/). Differentially expressed TEs were identified using edgeR (v3.38.4) following the criteria: FDR < 0.05 and |Log2 fold-change | > 0. The volcano plots were generated using EnhancedVolcano (v1.14.0).

### Protein extraction, antibodies, and immunoblotting

Whole-cell protein was extracted in 1× Laemmli Sample Buffer (Bio-Rad) and 1× Halt Protease and Phosphatase Inhibitor Cocktail (Thermo Fisher Scientific), prepared with 50 mM Tris (2-carboxyethyl) phosphine hydrochloride (TCEP-HCl; Thermo Fisher Scientific), separated by sodium dodecyl sulfate polyacrylamide gel electrophoresis (SDS-PAGE) and probed with primary antibodies (rabbit polyclonal anti-GNAS, Proteintech, #10150-2-AP, 1:1000; mouse monoclonal anti-histone H3, Cell Signaling Technology, #3638, 1:1500; rabbit monoclonal anti-histone H3K27ac, Cell Signaling Technology, #8173, 1:1500; mouse monoclonal anti-TBK1, Cell Signaling Technology, #51872, 1:1500; rabbit monoclonal anti-pTBK1 (S172), Cell Signaling Technology, #5483, 1:1000; mouse monoclonal anti-IRF3, Santa Cruz, #sc-33641, 1:500; rabbit monoclonal anti-pIRF3 (S386), Cell Signaling Technology, #37829, 1:1000; rabbit monoclonal anti-pCREB (S133), Abcam, #ab32096, 1:1000; mouse monoclonal anti-HDAC3, Cell Signaling Technology, #3949, 1:1000; mouse polyclonal anti-HDAC1, Cell Signaling Technology, #2062, 1:1000; mouse monoclonal anti-β-actin, Santa Cruz, #sc-47778, 1:500; mouse monoclonal anti-GAPDH, Cell Signaling Technology, #97166, 1:1500). For analysis of phospho-proteins, 20 mM NaF was added to blocking buffer (Intercept TBS Blocking Buffer, LI-COR) and antibody dilution buffer (blocking buffer + 0.1% Tween-20).

### T cell co-culture cytotoxicity assay

CD8^+^ T cells (negatively selected from freshly isolated primary human peripheral blood mononuclear cells) were obtained from BPS Bioscience, Inc. (CA, USA; #79753) and cultured at 1 × 10^6^ cells/mL in ImmunoCult-XF T cell Expansion Medium (STEMCELL Technologies; #10981) supplemented with 25 ng/mL human IL-2 (MedChem Express), 1% Non-Essential Amino Acids (Thermo Fisher Scientific) and 1% GlutaMAX Supplement (Thermo Fisher Scientific). T cells were activated with 25 µL/mL ImmunoCult Human CD3/CD28 T cell Activator (STEMCELL Technologies; #10971) for two days prior to co-culture with DLBCL cell lines. SU-DHL-4 cells with sgLacZ or sgGNAS3 were treated with 5 µM RGFP966 or DMSO for three days and stained with 1 µM 5-(and 6)-Carboxyfluorescein diacetate succinimidyl ester (CFSE; CFSE Cell Division Tracker Kit, BioLegend) for 20 min at room temperature in the dark immediately before co-culture with T cells. Stained SU-DHL-4 cells were then washed and resuspended in fresh warm culture media with or without activated CD8^+^ T cells at a ratio of 1:20. Twenty-four h after the beginning of co-culture, cells were collected, washed with cold PBS and resuspended in FACS buffer (PBS + 1% FBS) and then stained by 3 µM propidium iodide (PI, Thermo Fisher Scientific) for 15 min on ice in the dark. Samples were acquired by flow cytometry (FACSLyric Cell Analyzer, BD Biosciences). The total number of single DLBCL cells acquired per sample was 10,000. Data acquisition was performed using FACSuite software and analysis was performed using FlowJo software (v10.8.1; FlowJo).

### Immunofluorescence and epifluorescence imaging

Cells were treated with RGFP966 (1 µM for A20 or 5 µM for SU-DHL-4) or DMSO for five days. For positive control, cells were treated with 5-AZA-CdR (300 nM) for two days. Approximately 20,000 cells in PBS were deposited evenly onto charged slides (SuperFrost, Fisher Scientific) using a StatSpin Cytofuge 12 cytocentrifuge (Beckman Coulter) at 700 rpm for 10 min and allowed to dry at room temperature for 10 min. Cells on slides were then fixed in 4% ice-cold PFA for 10 min, washed in PBS plus 0.02% Tween-20 (PBST), and permeabilized in PBS plus 0.5% Tween-20 for 10 min. After permeabilization, slides were blocked with PBST plus 5% bovine serum albumin (BSA) at room temperature for 30 min. To visualize dsRNA, cells were stained with mouse anti-dsRNA J2 antibody (2.5 μg/ml, Scicons, #10010200) in PBST plus 2% BSA at room temperature for 1.5 h, washed in PBST for three times, and incubated with secondary goat anti-mouse Alexa Fluor 488 conjugated IgG (1:1000, Invitrogen) in PBST plus 2% BSA for 45 min. Cells were then washed in PBST three times, incubated with Hoechst 33342 to stain nuclei (1:1000, Thermo Fisher Scientific), and mounted using ProLong Gold antifade reagent (Thermo Fisher Scientific) with #1 cover glasses (VWR). Imaging and photography of cells were performed using an EVOS epifluorescence microscope (Thermo Fisher Scientific) using the GFP and DAPI channels with excitation at 488 nm and 405 nm, respectively. dsRNA was quantified by measuring integrated J2 density per cell using ImageJ (v1.53t).

### Analysis of DLBCL patient transcriptomic data

For the bulk RNA-seq dataset of DLBCL patients used in Ennishi et al. [36], the raw BAM files of a total of 376 transcriptome samples were downloaded from the European Genome Archive (EGA) repository (EGAD00001003783; https://ega-archive.org/datasets/EGAD00001003783). The raw BAM files were first converted into the FASTQ format using the SamToFastq tool, followed by a trimming/cleaning step using Trimmomatic (v0.39). The clean reads were mapped to a reference genome (GRCh37) and processed under the same workflow as for the cell-line bulk RNA-seq dataset. A final list of samples (*n* = 322) used for downstream analysis was determined according to Ennishi et al. [36]. For the bulk RNA-seq dataset of DLBCL patients used in Schmitz et al. [43], a total of 481 DLBCL transcriptome cases in the TSV format for the gene counts were downloaded from the Genomic Data Commons Data Portal (https://portal.gdc.cancer.gov/projects/NCICCR-DLBCL) and then combined into a count matrix as the input for downstream analysis. TE expression was detected using TEtranscripts as described above. Differential gene/TE expression analysis was performed using the Wilcoxon rank-sum test [70] following the criteria: FDR < 0.05 and |Log2 fold-change | > 0.5 (for genes) and FDR < 0.05 and |Log2 fold-change | > 0 (for TEs). The volcano plots were generated using EnhancedVolcano (v1.14.0). Pathway enrichment analysis was performed using g:Profiler or GSEA as described above.

### Statistical analysis

Statistical analyses were performed using R functions or packages. Only the results obtained from independent experiments (*n* ≥ 2) were included in the statistical analysis. All error bars represent ± standard error of the mean (SEM). For analysis of response ratios or normalized ratios, data were log2-transformed because the assumption was made that Log2 ratios were normally distributed if the ratios were not initially normally distributed after assessed by the Anderson-Darling normality test. Significantly differential results from bulk RNA-Seq and ATAC-seq analyses were obtained using the criteria: FDR < 0.05 and |Log2 fold-change | > 0, 0.5 or 1. For data following normal distribution, statistical significance was determined by an unpaired Welch *t*-test for two-group comparison or one-way analysis of variance (ANOVA) for multiple comparisons (Tukey’s HSD test performed with adjusted *P*-values reported). For data not following normal distribution, statistical significance was determined by a Wilcoxon rank-sum test for two-group comparison or a Kruskal-Wallis test for multiple comparisons (pairwise Wilcoxon rank-sum test performed with *P*-values reported). For all statistical analyses, a *P*-value or adjusted *P*-value < 0.05 was considered sufficient to reject the null hypothesis.

## Supplementary information

Descriptions of Supplementary Data

Supplementary Information

Supplementary Data 1

Supplementary Data 2

Supplementary Data 3

Supplementary Data 4

Supplementary Data 5

## Data Availability

The cell-line bulk RNA-Seq dataset has been deposited in Gene Expression Omnibus (GEO) with the accession number GSE268034. Relevant data are available in the article, Supplementary Information and Supplementary Data. The full unedited blots for immunoblotting analysis were included in Supplementary Figs. 10–14.
