# Supplementary material for: GNAS knockout potentiates HDAC3 inhibition through viral mimicry-related interferon responses in lymphoma
Source: Leukemia. 2024 Aug 8;38(10):2210–24. doi: 10.1038/s41375-024-02325-4 (PMC11436380; doi:10.1038/s41375-024-02325-4)
Supplement: Supplementary file 1 — Descriptions of Supplementary Data [file 41375_2024_2325_MOESM1_ESM.pdf]

Description of Supplementary Data

Supplementary Data 1.
Analysis of *CREBBP* mutations in DLBCL cell lines.

Supplementary Data 2.
MAGeCK analysis of the genome-wide CRISPR screen, showing the gene ranking lists for the comparisons between (1) drug treatment versus DMSO on day 14 and (2) DMSO on day 14 versus day 0.

Supplementary Data 3.
Differentially expressed gene analysis of cell-line bulk RNA-seq data across different comparisons and the full gene list from clustering analysis.

Supplementary Data 4.
Gene set enrichment analysis of cell-line bulk RNA-seq data across different comparisons using the H collection of hallmark gene sets (MSigDB).

Supplementary Data 5.
Gene set enrichment analysis of patient sample bulk (*GNAS* high versus *GNAS* low) or cell-line bulk (LacZ versus *GNAS* KO) RNA-seq data using the C5 GO subcollection of Gene Ontology gene sets (MSigDB).
