# Supplementary material for: GNAS knockout potentiates HDAC3 inhibition through viral mimicry-related interferon responses in lymphoma
Source: Leukemia. 2024 Aug 8;38(10):2210–24. doi: 10.1038/s41375-024-02325-4 (PMC11436380; doi:10.1038/s41375-024-02325-4)
Supplement: Supplementary file 2 — Supplementary Information [file 41375_2024_2325_MOESM2_ESM.pdf]

GNAS knockout potentiates HDAC3 inhibition through viral mimicry-related interferon responses in lymphoma

Michael Y. He[1], Kit I. Tong[1], Ting Liu[1], Ryder Whittaker Hawkins[2], Victoria Shelton[1], Yong Zeng[1], Mehran Bakhtiari[1], Yufeng Xiao[3], Guangrong Zheng[3], Ali Sakhdari[4], Lin Yang[1,5], Wenxi Xu[1], David G. Brooks[1,2], Rob C. Laister[1], Housheng Hansen He[1,5], Robert Kridel[1,5,6,7*]

[1] Princess Margaret Cancer Centre, University Health Network, Toronto, ON, Canada

[2] Department of Immunology, University of Toronto, Toronto, ON, Canada

[3] Department of Medicinal Chemistry, College of Pharmacy, University of Florida, Gainesville, FL, USA

[4] Laboratory Medicine and Pathobiology, University Health Network, Toronto, ON, Canada

[5] Department of Medical Biophysics, University of Toronto, Toronto, ON, Canada

[6] Institute of Medical Science, University of Toronto, Toronto, ON, Canada

[7] Department of Medicine, University of Toronto, Toronto, ON, Canada

Supplementary Information

Supplementary Methods

Targeted DNA sequencing and cell line mutation analysis

Genomic DNA was isolated using DNeasy Blood and Tissue Kit (QIAGEN) according to the manufacturer's instructions. Library construction and sequencing were performed by the GSC in Vancouver, BC. Methods related to targeted sequencing of the PLOSMED are described in Kridel and Chan et al.[1]. BAM files were analyzed in tumor-only mode by Mutect2 (GATK v4.1.8.1)[2] to obtain a list of single nucleotide variants (SNVs) and insertions and deletions (indels). Interval files were prepared using targeted probe coordinates and the GATK function BedToIntervalList. Targeted sequencing data were aligned to the GRCh37 reference human genome. Unmapped reads, PCR duplicates, and off-target variants were removed. CollectTargetedPcrMetrics was utilized to extract per-target coverage where downstream assessment and filtering included a threshold of 50× mean sample coverage. The mean on-target coverage of the total cohort was 413× (± 340×). GnomAD raw sites population variants (af-only-gnomad.raw.sites.b37.vcf.gz, downloaded June 25th, 2019)[3] were used as the germline resource. GATK FilterMutectCalls was used to filter raw mutation calls. Variant VCF files were then normalized using Vt (v0.577)[4] and duplicates were removed. Variant annotation was achieved using Annovar (20180416)[5]. Post-processing was carried out to discard variants that did not fulfill the following criteria:

- Variant achieves a 'PASS' from GATK FilterMutectCalls.
- Variant does not overlap a single nucleotide polymorphism (SNP) (avsnp142/dbSNP 142[6]); *OR* if the variant does overlap an SNP this variant is also annotated by COSMIC (cosmic68[7]).
- Variant with an allele fraction > 0.1.
- Variant with approximate read depth greater than 10.
- The variant has population variant frequency less than 0.001 (gnomad211_genome, gnomAD genome collection (v2.1.1))[3].

Lentivector preparation and transduction

To prepare lentivectors (LVs), HEK293T cells were co-transfected with the LV transfer plasmid Lenti-Cas9-2A-Blast (RRID:Addgene_73310) or pLCKO (RRID:Addgene_73311), the LV packaging plasmid psPAX2 (RRID:Addgene_12260) and the envelope-encoding plasmid pMD2.G (RRID:Addgene_12259) at a mass ratio of 10:9:1, respectively, using Lipofectamine 2000 (Thermo Fisher Scientific) according to manufacturer's instruction. LV collection medium (DMEM + 1.1% albumin) supernatant containing LVs was filtered and collected at 48 h and 72 h post-transfection and stored at –80 ºC. Lymphoma cells were transduced with LV suspension in culture media plus 4.8 µg/mL polybrene.

Generation of KO cell lines using CRISPR–Cas9

To construct plasmids, oligonucleotide duplexes (ordered from Integrated DNA Technologies Inc.) were inserted into pLCKO via the BfuAI (New England BioLab, NEB) digested site using T4 ligase (NEB) and transformed into one shot TOP10 chemically competent *E. coli* (Thermo Fisher Scientific) according to the manufacturer's instructions. Positive clones were selected using ampicillin and ampicillin-resistant cells were then screened by Sanger sequencing for positive constructs.

SU-DHL-4, 5, 10–Cas9 or A20–Cas9 cells (lymphoma cells expressing Cas9 generated as previously described[8]) were transduced with LVs encoding sgRNAs expressed under a U6 promoter. Positively transduced cells were then selected using puromycin for three days. To generate the negative control cells,

LVs encoding sgRNA targeting the *LacZ* gene were introduced into SU-DHL-4, 5, 10–Cas9 or A20–Cas9 cells. The sgRNA sequences are:

GNAS (sgRNA1; sgGNAS1): 5'-GATCCTCATCTGCTTCACAA-3'
GNAS (sgRNA2; sgGNAS2): 5'-TGAGAAGGCAACCAAAGTGC-3'
GNAS (sgRNA3; sgGNAS3): 5'-GGGTTGGCCAGCTCCACGGG-3'
LacZ (sgLacZ): 5'-CAGCTGGCGTAATAGCGAAG-3'

cAMP detection

Cells were treated with RGFP966 (5 µM) or DMSO for five days. For each sample, cells were collected, washed with cold PBS, and resuspended in Induction Buffer (500 µM 3-Isobutyl-1-methylxanthine (IBMX; Sigma) and 100 µM 4-(3-Butoxy-4-methoxybenzyl)imidazolidin-2-one (Ro 20-1724; Sigma), two cAMP phosphodiesterase inhibitors, in PBS) at 500,000/mL in 96-well white flat-bottom plates (Greiner Bio-One). Released cAMP levels were detected using the cAMP-Glo Assay (Promega, #V1501) according to the manufacturer's instructions. The cAMP analog 8-Br-cAMP was added as a positive control. Luminescence was measured using the Spectramax M5 microplate reader (Molecular Devices).

Apoptosis assay

Cells were treated with RGFP966 (5 µM) or DMSO for five days. One million cells per condition were collected, washed with cold PBS twice, resuspended in 1× Annexin V Binding Buffer, and then stained by Annexin V–APC (BD Biosciences, #550475) and/or PI (Bioscience, #00699050) according to the manufacturer's instructions. Samples were acquired by flow cytometry (LSRFortessa Cell Analyzer, BD Biosciences). The total number of single cells acquired per sample was 10,000. Data acquisition was performed using FACSDiva software and analysis was performed using FlowJo software (v10.8.1; FlowJo).

Nuclear DNA content analysis

Cells were treated with RGFP966 (5 µM) or DMSO for five days. Two million cells per condition were collected, washed with cold PBS twice, resuspended in 50 µL PBS, and added to 1 mL of ice-cold 80% ethanol at 4 ºC. Fixed cells were washed with cold PBS once, resuspended in 500 µL of 2 mg/mL RNase A (QIAGEN) in PBS, and incubated at room temperature for 5 min. Subsequently, 500 µL of 0.1 mg/mL PI solution was added and the sample was incubated at room temperature for 30 min in the dark. Samples were acquired by flow cytometry (LSRFortessa Cell Analyzer). The total number of single cells acquired per sample was 10,000. Data acquisition was performed using FACSDiva software and analysis was performed using FlowJo software.

Cell-line bulk ATAC-seq and analysis

SU-DHL-4 cells were treated with RGFP966 (5 µM) or DMSO for five days. For each sample, 60,000 viable (viability > 80%) cells were pelleted at 500 × *g* for 5 min at 4 ºC, resuspended in 50 µL of fully supplemented ATAC-seq resuspension buffer (RSB) containing 0.1% NP-40, 0.1% Tween-20, and 0.01% digitonin, and incubated on ice for 3 min. Following lysis, 1 mL of ATAC-seq RSB containing 0.1% Tween-20 was added to stop the lysis. Nuclei were centrifuged at 500 × *g* for 10 min at 4 ºC and resuspended in 50 µL of transposition master mix (25 µL 2× TD buffer, 2.5 µL transposase, 16.5 µL PBS, 0.5 µL 1% digitonin, 0.5 µL 10% Tween-20, and 5 µL water). Transposition reactions were incubated at 37 ºC for 30 min in a thermomixer with shaking at 1,000 rpm. Reactions were cleaned up with MinElute columns (QIAGEN, #28204) and the purified DNA fragments were amplified using NEB Next HiFi 2× PCR mix with the ATAC Ad primers (Ad1 universal primer and Ad2.X sequencing primer). The following thermocycling parameters were used: 72 ºC for 5 min, 98 ºC for 30 s, 20 cycles of (98 ºC for 10 s, 63 ºC

for 30 s, 72 ºC for 1 min). PCR product purification and size selection were achieved using AMPure XP Beads (Beckman Coulter, #A63882). The final libraries were run on the Agilent 2100 Bioanalyzer High Sensitivity DNA Chip to check the library size before sequencing. The samples were sequenced PE50 on Illumina's NovaSeq 6000 using an S1 flowcell to achieve a read depth of 50–60 million reads per sample. For the ATAC-seq data, the quality control, alignment to the human genome hg38, and peak calling were executed employing the ENCODE ATAC-seq pipeline (v1.10.0, https://www.encodeproject.org/atac-seq/). Subsequently, differentially accessible sites (DASs) were discerned and annotated using DiffBind (v3.8.4)[9] and ChIPseeker (v1.34.1)[10], respectively. These analyses were conducted based on the conventional peaks that surpassed the irreproducible discovery rate threshold across the replicates for each condition. Lastly, DASs were intersected with TEs based on the TE annotation (the curated TE GTF file was downloaded from https://labshare.cshl.edu/shares/mhammelllab/www-data/TEtranscripts/TE_GTF/).

Motif enrichment analysis

Following ATAC-seq peak calling, significantly upregulated peaks (FDR < 0.05) were used for analyzing motif enrichment using HOMER (v4.11)[11] with the parameter "findMotifsGenome.pl hg38 -size 200 -mask", which allows HOMER to find motifs within +/– 100 bp from the peak center. The Log2 fold change was calculated as *log2FC = log2(Pti/Pbi)*, where *Pti* refers to the percentage of the target sequence with motif *i*, and *Pbi* refers to the percentage of the background sequence with motif *i*, as estimated by HOMER.

Integrated analysis of ATAC-seq and RNA-seq

Differentially upregulated genes in GNASKO3+RGFP966 were identified from cell-line bulk RNA-seq analysis (FDR < 0.05). Functional enrichment analysis was performed to infer transcription factor activity based on the differentially upregulated genes using the TRRUST database[12] in Enrichr[13]. The top enriched transcription factors from RNA-seq were compared with the unique transcription factors/motifs enriched in GNASKO3+RGFP966 from ATAC-seq.

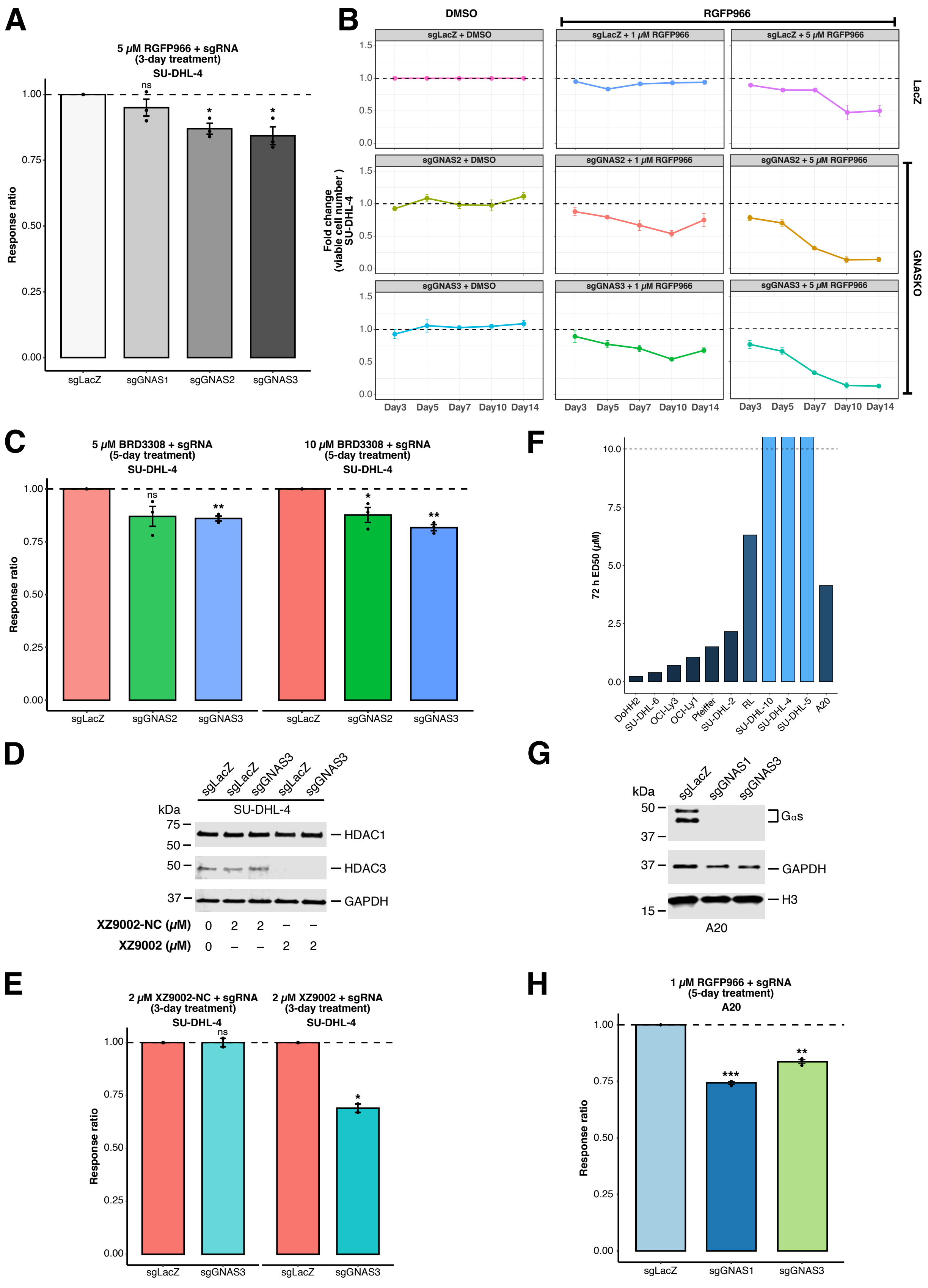
A
5 μM RGFP966 + sgRNA
(3-day treatment)
SU-DHL-4
Response ratio
ns
*
*
sgLacZ
sgGNAS1
sgGNAS2
sgGNAS3
B
DMSO
RGFP966
sgLacZ + DMSO
sgLacZ + 1 μM RGFP966
sgLacZ + 5 μM RGFP966
sgGNAS2 + DMSO
sgGNAS2 + 1 μM RGFP966
sgGNAS2 + 5 μM RGFP966
sgGNAS3 + DMSO
sgGNAS3 + 1 μM RGFP966
sgGNAS3 + 5 μM RGFP966
Fold change
(viable cell number)
SU-DHL-4
LacZ
GNASKO
Day3
Day5
Day7
Day10
Day14
C
5 μM BRD3308 + sgRNA
(5-day treatment)
SU-DHL-4
10 μM BRD3308 + sgRNA
(5-day treatment)
SU-DHL-4
Response ratio
ns
**
*
**
sgLacZ
sgGNAS2
sgGNAS3
D
sgLacZ
sgLacZ
sgGNAS3
sgLacZ
sgGNAS3
SU-DHL-4
kDa
HDAC1
HDAC3
GAPDH
XZ9002-NC (μM)
XZ9002 (μM)
E
2 μM XZ9002-NC + sgRNA
(3-day treatment)
SU-DHL-4
2 μM XZ9002 + sgRNA
(3-day treatment)
SU-DHL-4
Response ratio
ns
*
sgLacZ
sgGNAS3
F
72 h ED50 (μM)
DoHH2
SU-DHL-6
OCI-Ly3
OCI-Ly1
Pfeiffer
SU-DHL-2
RL
SU-DHL-10
SU-DHL-4
SU-DHL-5
A20
G
kDa
sgLacZ
sgGNAS1
sgGNAS3
Gαs
GAPDH
H3
A20
H
1 μM RGFP966 + sgRNA
(5-day treatment)
A20
Response ratio
***
**
sgLacZ
sgGNAS1
sgGNAS3

Supplementary Figure 1. Additional validation of *GNAS* KO-induced sensitization in human and mouse lymphoma cell lines using selective HDAC3 inhibitors or HDAC3-specific degrader.
(A) Effects of three sgRNAs targeting *GNAS* (sgGNAS1, 2, 3) in SU-DHL-4 cells treated with RGFP966 over three days. sgGNAS2 and sgGNAS3 showed significant sensitizing effects while sgGNAS1 showed a trend of sensitization. **(B)** *GNAS* KO sensitized SU-DHL-4 cells to RGFP966 without causing inhibitory effects on survival and/or proliferation. All values are normalized versus sgLacZ + DMSO and shown as fold change of viable cell numbers over 14 days of cell culture. **(C)** Effects of sgGNAS2 and sgGNAS3 in SU-DHL-4 cells treated with BRD3308 over five days. sgGNAS3 showed significant sensitizing effects at both doses tested while sgGNAS2 showed significant sensitizing effects at 10 μM. **(D)** Western blotting analysis of HDAC3 expression showed effective and specific degradation under treatment with HDAC3-specific degrader XZ9002 but not XZ9002-NC (a negative control compound of XZ9002 with an inactive VHL ligand) in SU-DHL-4 cells over three days. Control LacZ cells treated with DMSO were used to indicate baseline expression. HDAC1 was included to indicate potential off-target effects of XZ9002. **(E)** Effects of sgGNAS3 in SU-DHL-4 cells treated with XZ9002 and XZ9002-NC over three days. sgGNAS3 showed significant sensitizing effects in cells treated with XZ9002 but not XZ9002-NC. **(F)** Response of the mouse lymphoma cell line A20 to HDAC3 inhibitor RGFP966. Responses of the 10 DLBCL cell lines were included for reference. **(G)** Western blotting analysis of Gαs expression in A20 cells upon CRISPR–Cas9 gene editing. **(H)** Effects of sgGNAS1 and sgGNAS3 in A20 cells treated with RGFP966 over five days. Both sgGNAS1 and sgGNAS3 showed significant sensitizing effects. See Methods and Fig. 1 F and H for details of the calculation of the response ratios. Error bars represent ± SEM in A–C, E, H (n = 3 independent experiments except for results in B and E where n = 2). *, $p < 0.05$; **, $p < 0.01$; ***, $p < 0.001$, ns, not significant (Log2 transformed ratios versus Log2(1) in A, C, E, H; one-way ANOVA with Tukey's HSD test for multiple comparisons).

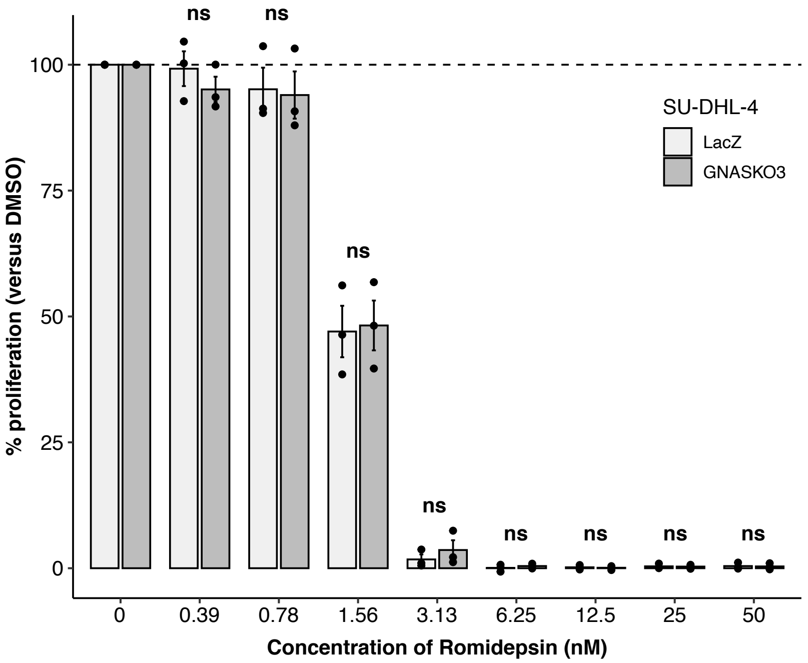

Supplementary Figure 2. *GNAS* KO does not sensitize SU-DHL-4 cells to the pan-HDAC inhibitor romidepsin.

SU-DHL-4 cells with sgLacZ or sgGNAS3 were treated with DMSO or the pan-HDAC inhibitor romidepsin at the indicated doses for 72 h. Cell viability was measured using alamarBlue reagents. Percentage proliferation was calculated by normalization versus DMSO. Error bars represent SEM (n = 3 independent experiments). ns, not significant (log2 transformed values for each dose were compared; two-tailed unpaired Welch's t-test).

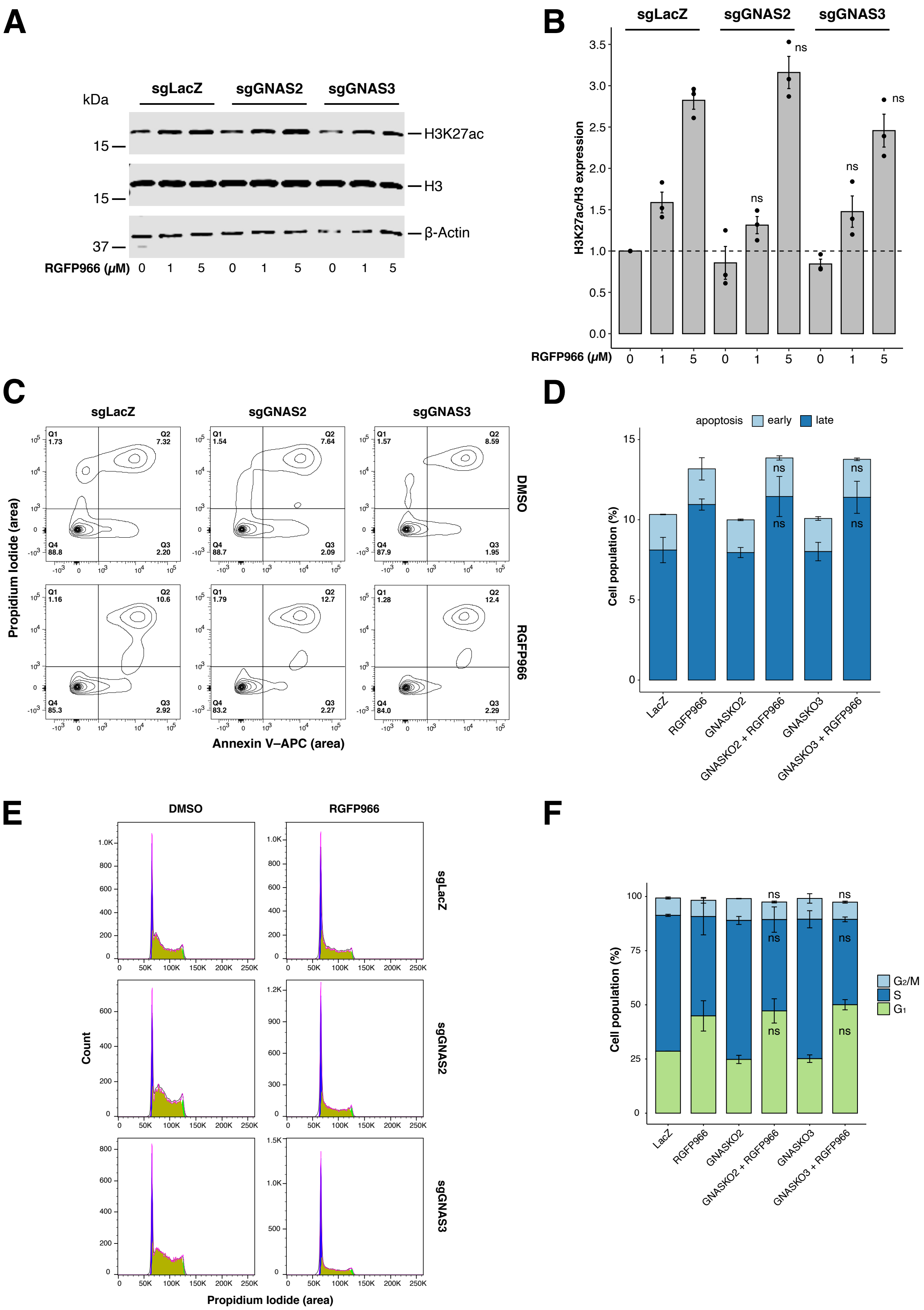

A
kDa
sgLacZ
sgGNAS2
sgGNAS3
H3K27ac
H3
β-Actin
15
15
37
RGFP966 (μM) 0 1 5 0 1 5 0 1 5
B
sgLacZ
sgGNAS2
sgGNAS3
H3K27ac/H3 expression
ns
RGFP966 (μM) 0 1 5 0 1 5 0 1 5
C
sgLacZ
sgGNAS2
sgGNAS3
DMSO
RGFP966
Propidium Iodide (area)
Annexin V–APC (area)
D
apoptosis
early
late
Cell population (%)
LacZ
RGFP966
GNASKO2
GNASKO2 + RGFP966
GNASKO3
GNASKO3 + RGFP966
E
DMSO
RGFP966
sgLacZ
sgGNAS2
sgGNAS3
Count
Propidium Iodide (area)
F
Cell population (%)
G2/M
S
G1
LacZ
RGFP966
GNASKO2
GNASKO2 + RGFP966
GNASKO3
GNASKO3 + RGFP966

Supplementary Figure 3. *GNAS* KO does not affect HDAC3 inhibition-induced acetylation, apoptosis, or cell cycle arrest.
(A–B) Western blotting analysis of H3K27 acetylation in SU-DHL-4 cells. Cells were treated for five days before lysis. **(C–D)** Effects of RGFP966 and/or *GNAS* KO on apoptosis in SU-DHL-4 cells. Early and late apoptosis were defined by Annexin V-APC^{+}/Propidium Iodide^{-} (Q3) and Annexin V-APC^{+}/Propidium Iodide^{+} (Q2), respectively. **(E–F)** Effects of RGFP966 and/or *GNAS* KO on cell cycle progression in SU-DHL-4 cells. Error bars represent ± SEM (n = 2 independent experiments except for Western blotting experiments where n = 3). All comparisons were made between GNASKO(2 or 3)+RGFP966 and RGFP966. ns, not significant (Log2 transformed ratios were compared; one-way ANOVA with Tukey's HSD test for multiple comparisons).

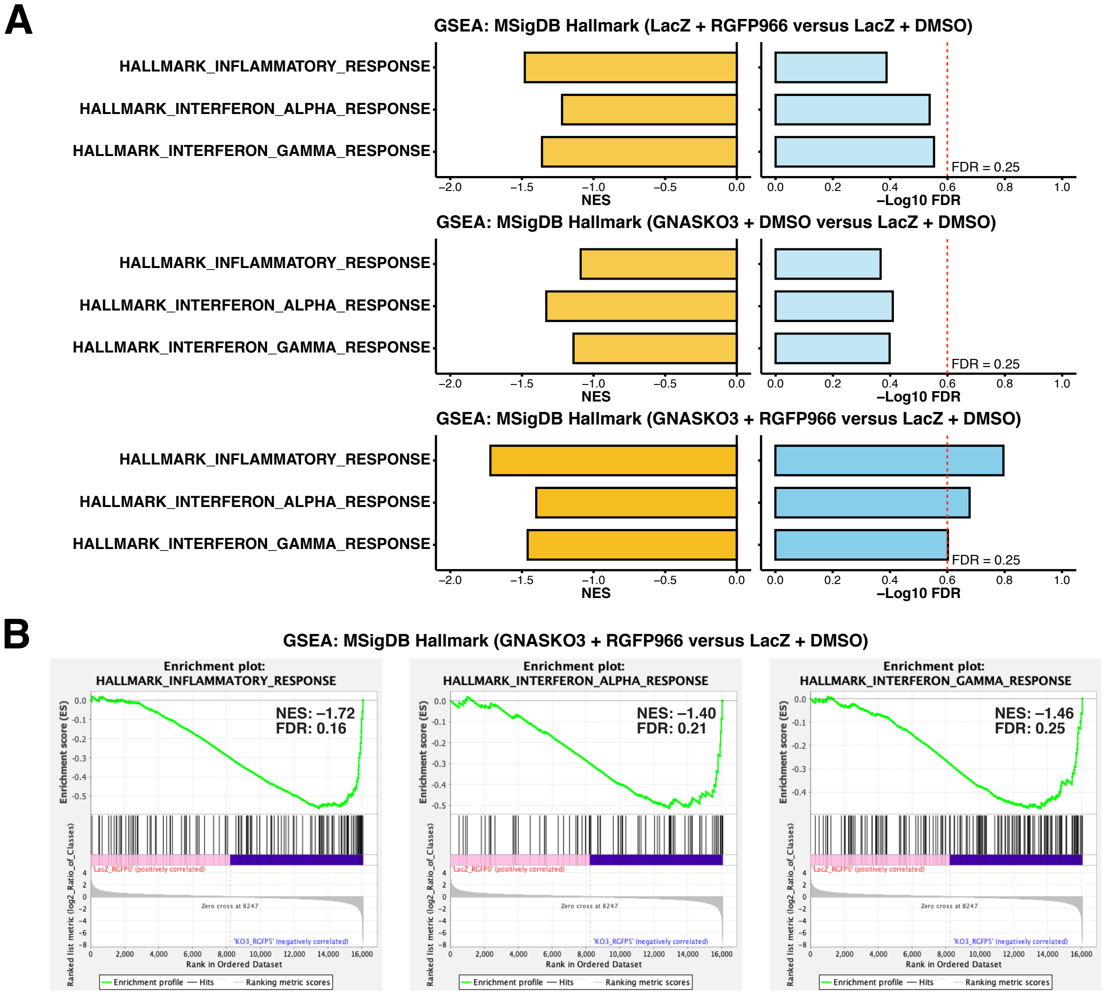

Supplementary Figure 4. Gene set enrichment analysis (GSEA) of the transcriptomic profiles in SU-DHL-4 cells shows activated interferon signaling in *GNAS* KO plus HDAC3 inhibition.
(A) Interferon response gene sets in SU-DHL-4 cells treated with RGFP966 (LacZ+RGFP966), *GNAS* KO SU-DHL-4 cells (GNASKO3+DMSO), and *GNAS* KO SU-DHL-4 cells treated with RGFP966 (GNASKO3+RGFP966). Red dotted lines indicate a cut-off of FDR = 0.25. **(B)** Enrichment plots of the gene set enriched in *GNAS* KO SU-DHL-4 cells treated with RGFP966 (GNASKO3+RGFP966). All interferon response gene sets were significantly enriched in GNASKO3+RGFP966. FDR, false discovery rate; NES, normalized enrichment score.

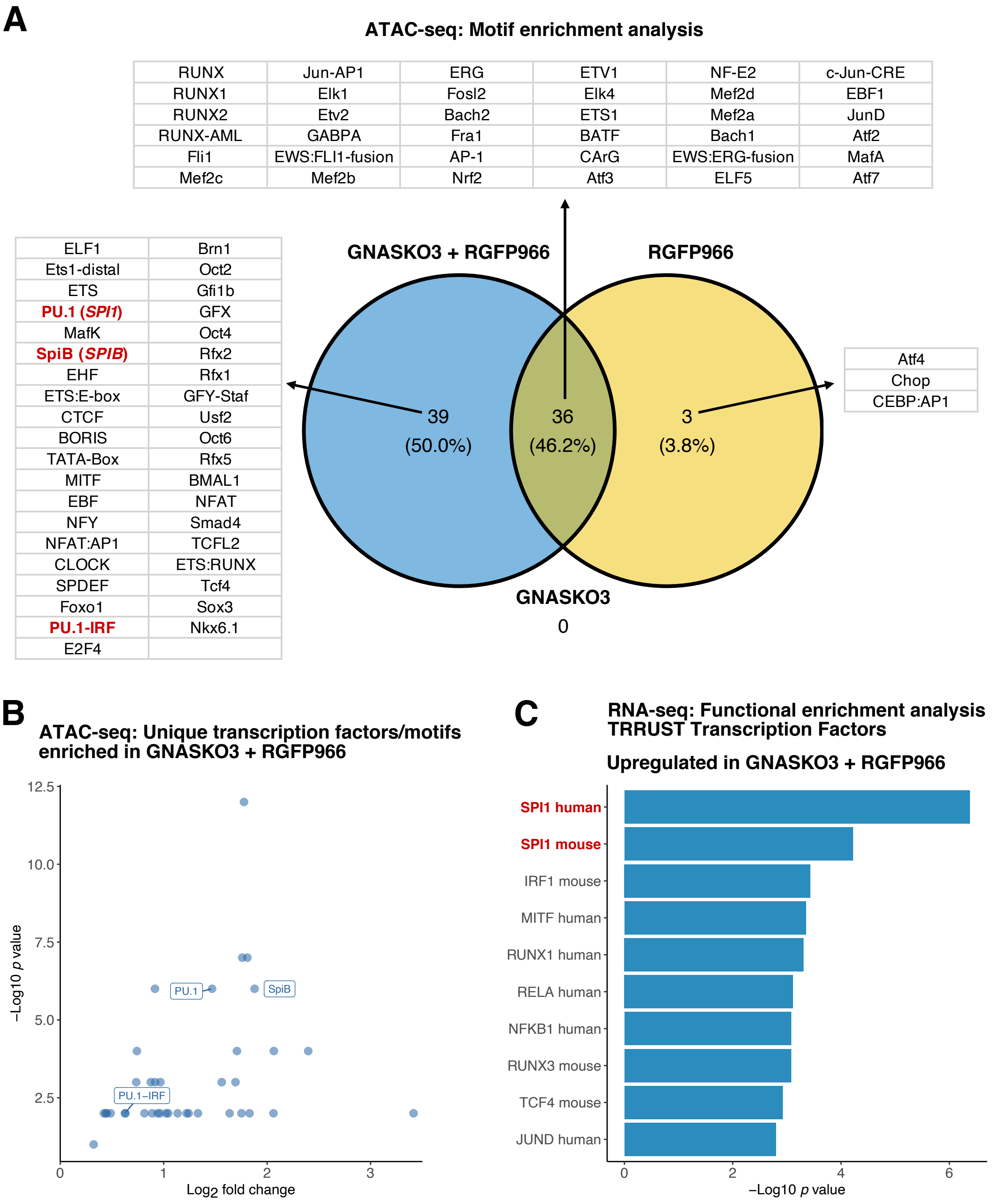

Supplementary Figure 5. Integrated analysis of ATAC-seq and RNA-seq in SU-DHL-4 cells. **(A)** Motif enrichment analysis of differentially upregulated peaks (identified from ATAC-seq) reveals potential motifs and the corresponding transcription factors enriched under each condition. **(B)** Unique transcription factors/motifs enriched in GNASKO3+RGFP966. **(C)** Functional enrichment analysis of differentially upregulated genes (identified from RNA-seq) shows top enriched transcription factors in GNASKO3+RGFP966 based on the TRRUST database. Common transcription factors are highlighted in red.

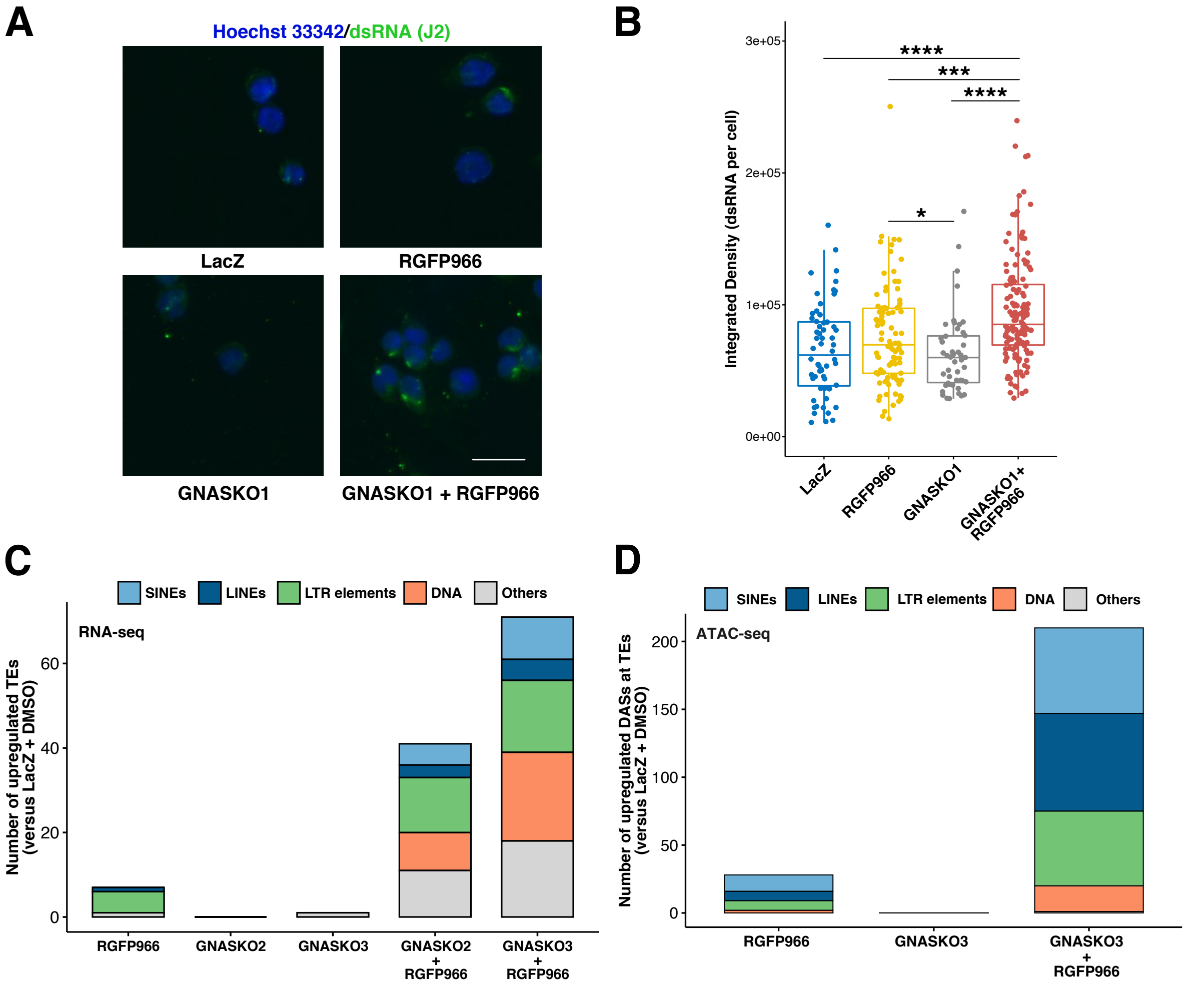

Supplementary Figure 6. *GNAS* KO plus RGFP966 is associated with dsRNA formation and TE expression (additional data).

(A) Representative images of A20 cells stained by Hoechst 33342 (for cell nuclei; blue) and J2 antibody (for dsRNAs; green) from immunofluorescence analysis. Scale bar, 25 µm. **(B)** Quantification of results in A. *, $p < 0.05$; ***, $p < 0.001$; ****, $p < 0.0001$ (Kruskal–Wallis test with Wilcoxon rank sum test for pairwise comparisons). **(C)** Upregulated TEs identified based on TE families from cell-line (SU-DHL-4) bulk RNA-seq data (FDR < 0.05 and Log2 fold change > 0). **(D)** Upregulated differential accessibility sites (DASs) at TEs identified based on TE loci from cell-line (SU-DHL-4) bulk ATAC-seq data (FDR < 0.05 and Log2 fold change > 1). Results represent two independent experiments ($n = 2$).

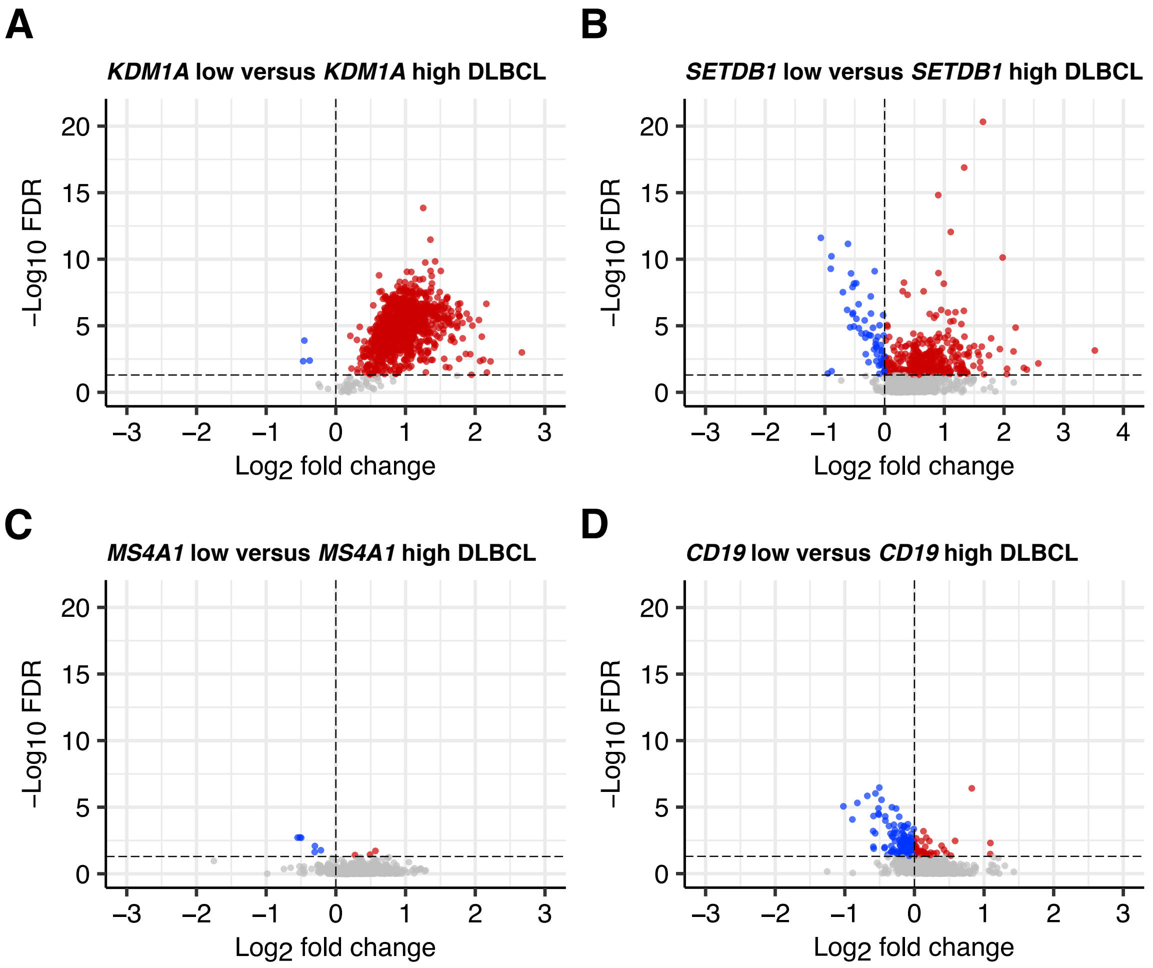

Supplementary Figure 7. Differential TE expression in DLBCL patients.
Volcano plots showing normalized expression dynamics of differentially expressed TEs (FDR < 0.05 and |Log2 fold change| > 0) identified from TEtranscripts analysis of DLBCL patient bulk RNA-seq data (Ennishi, et al 2019). Gene low and high groups were defined based on median expression in DLBCL patients. DLBCL samples were collected at diagnosis (Ennishi et al. 2019; $n = 322$). The comparison was made between **(A)** *KDM1A* low and high DLBCL patients, **(B)** *SETDB1* low and high DLBCL patients, **(C)** *MS4A1* low and high DLBCL patients, or **(D)** *CD19* low and high DLBCL patients. Upregulated and downregulated TEs are highlighted in red and blue, respectively. The horizontal and vertical dotted lines indicate FDR = 0.05 and Log2 fold change = 0, respectively.

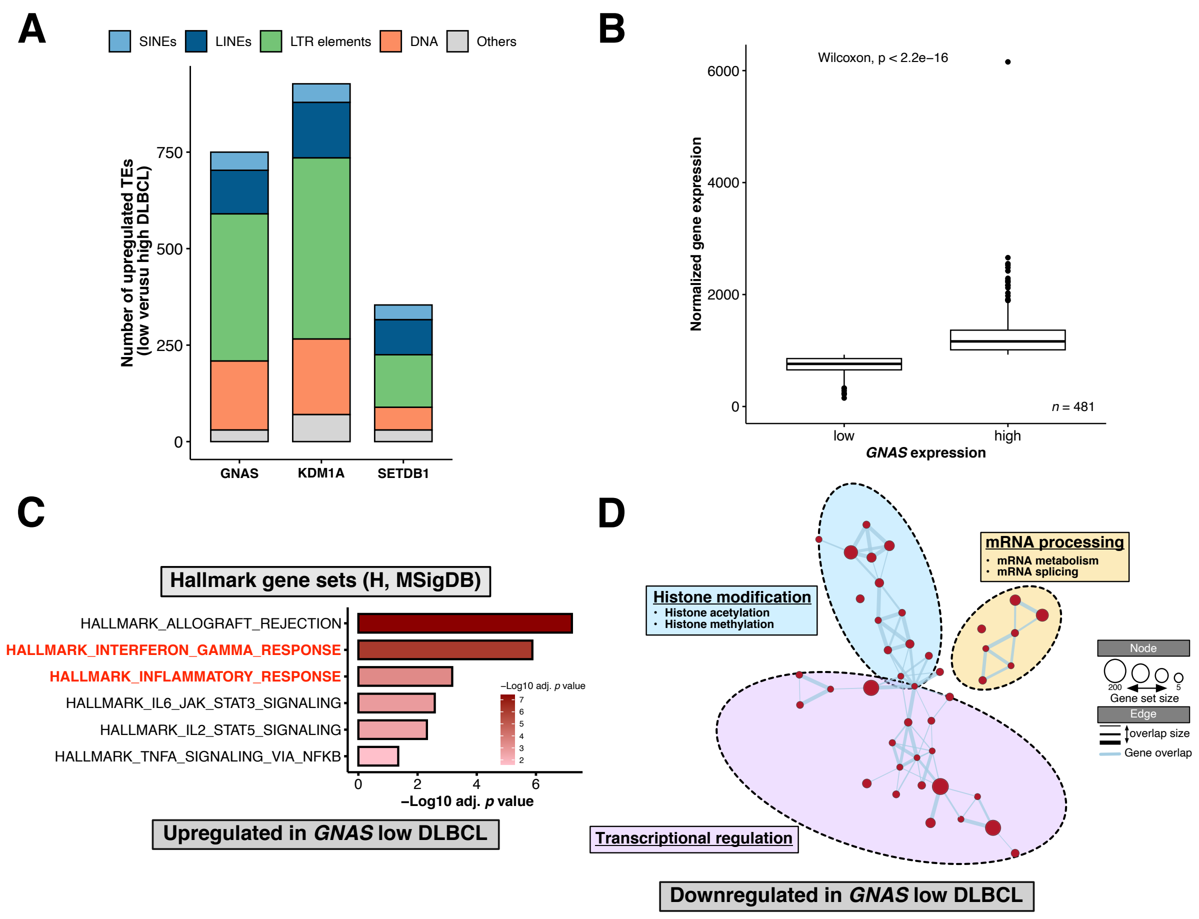

Supplementary Figure 8. Characterization of *GNAS* expression on TE expression and pathway enrichment in DLBCL patients (additional data).
(A) Upregulated TEs identified based on TE families from DLBCL patient bulk RNA-seq data (FDR < 0.05 and Log2 fold change > 0). **(B)** Classification of *GNAS* low and high groups based on median *GNAS* expression in DLBCL patients. DLBCL samples were collected at diagnosis (Schmitz et al. 2018; $n = 481$). **(C)** Pathway enrichment analysis of the genes upregulated in *GNAS* low DLBCL patients using the MSigDB H collection by g:Profiler. Significantly enriched gene sets (FDR < 0.05) are highlighted by –Log10 FDR and the interferon response gene sets are highlighted in red. **(D)** Cytoscape-generated enrichment maps showing gene network clusters based on GSEA using the MSigDB C5 GO subcollection (FDR < 0.3). GSEA was performed for the comparison between *GNAS* high versus low. Each node represents a gene set and links represent gene overlap between sets. Three major biological themes histone modification, mRNA processing, and transcriptional regulation are identified and highlighted. Results in A are based on data from Ennishi et al. 2019. Results in B–D are based on data from Schmitz et al. 2018.

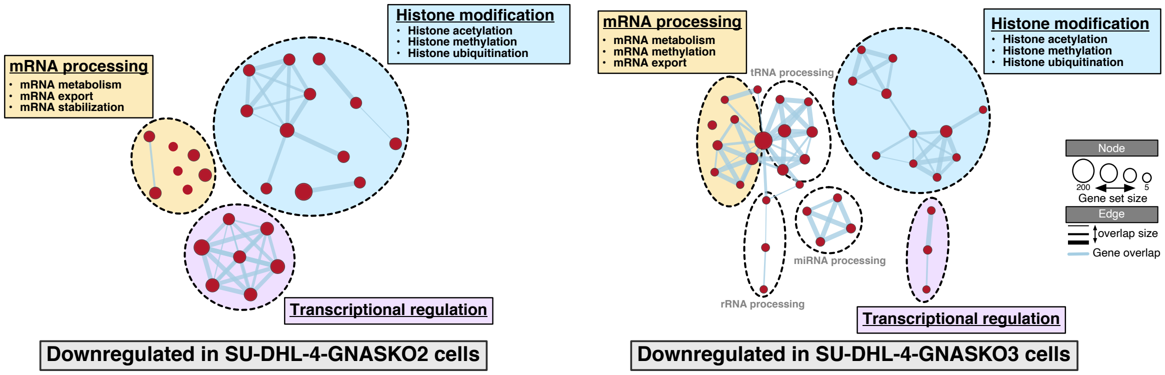

Supplementary Figure 9. Characterization of *GNAS* KO on gene set enrichment in DLBCL SU-DHL-4 cells with sgGNAS2 (left) or sgGNAS3 (right).

Cytoscape-generated enrichment maps showing gene network clusters based on GSEA using the MSigDB C5 GO subcollection (FDR < 0.3). GSEA was performed for the comparison between LacZ+DMSO versus GNASKO+DMSO. Each node represents a gene set and links represent gene overlap between sets. Three major biological themes histone modification, mRNA processing, and transcriptional regulation are identified and highlighted.

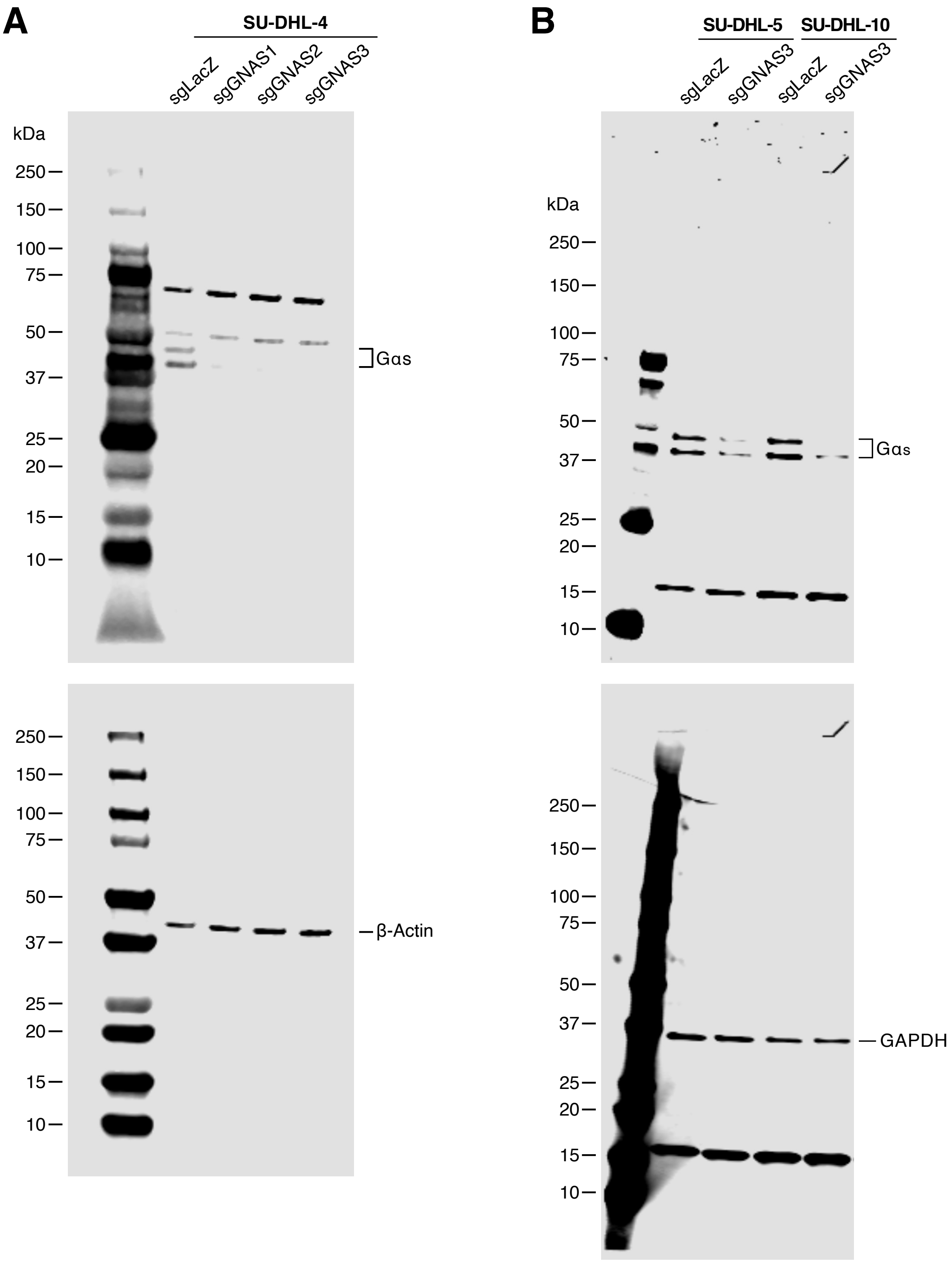

Supplementary Figure 10. Full unedited blots for Figure 1E (A) and Figure 1G (B).

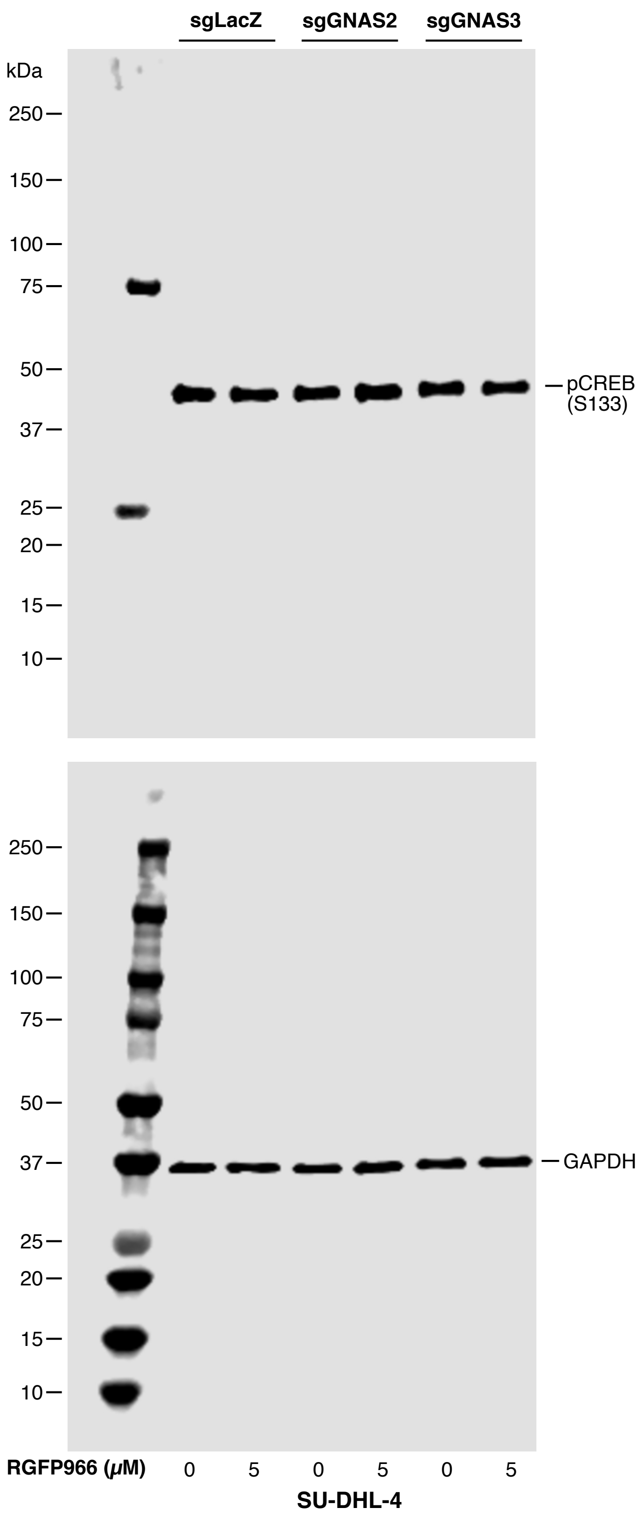

Supplementary Figure 11. Full unedited blots for Figure 2C.

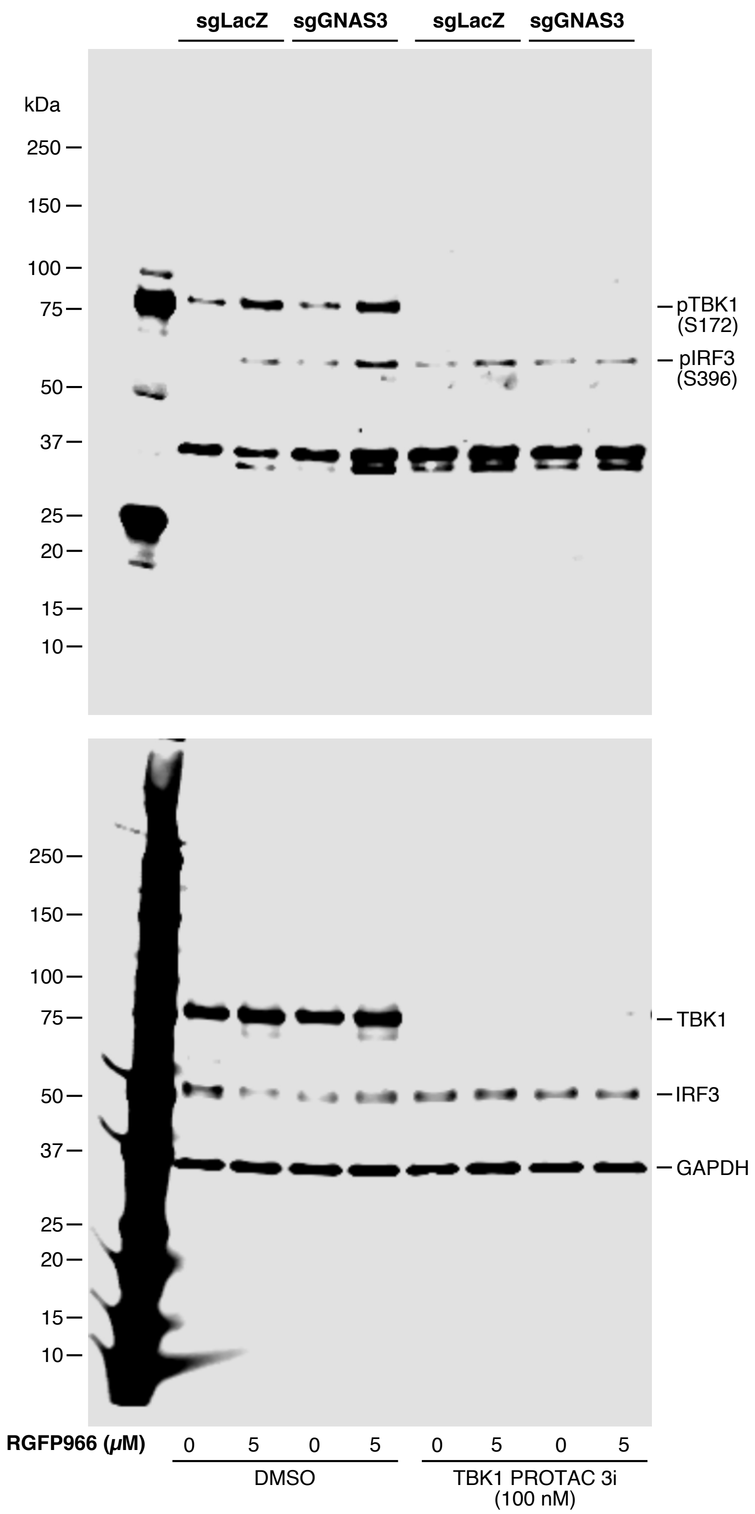

Supplementary Figure 12. Full unedited blots for Figure 3C.

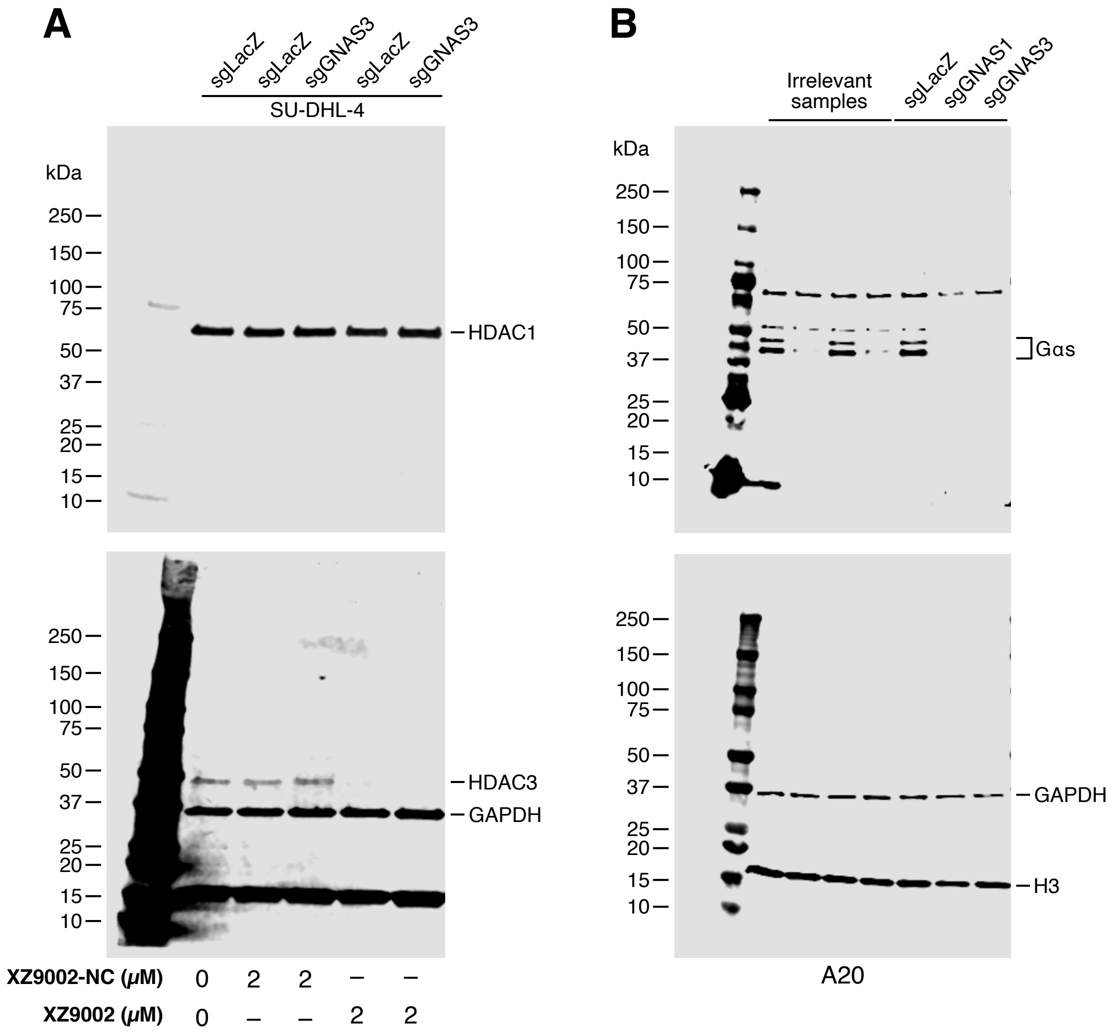

Supplementary Figure 13. Full unedited blots for Supplementary Figure 1D (A) and Supplementary Figure 1G (B).

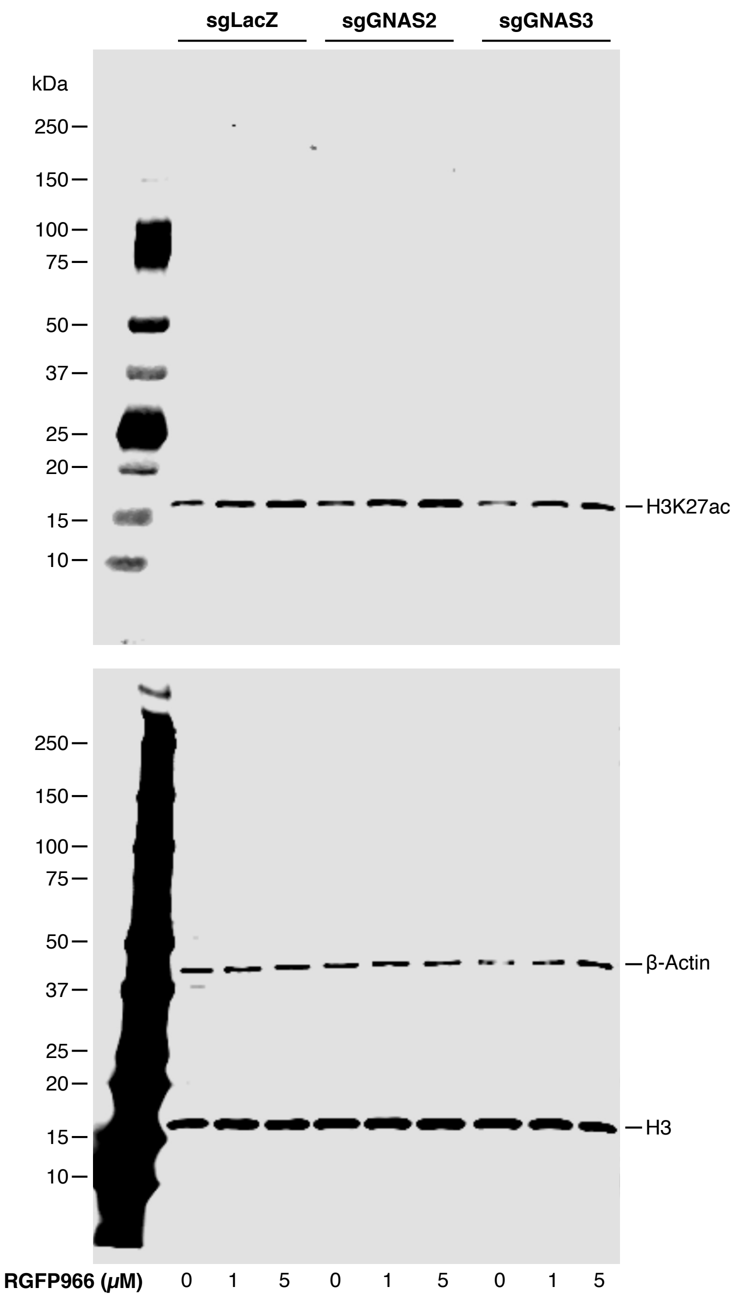

Supplementary Figure 14. Full unedited blots for Supplementary Figure 3A.

References

1 Kridel R, Chan FC, Mottok A, Boyle M, Farinha P, Tan K *et al.* Histological Transformation and Progression in Follicular Lymphoma: A Clonal Evolution Study. *PLoS Med* 2016; **13**: e1002197.

2 Van der Auwera GA, O'Connor BD. *Genomics in the Cloud: Using Docker, GATK, and WDL in Terra*. 'O'Reilly Media, Inc.', 2020.

3 Karczewski KJ, Francioli LC, Tiao G, Cummings BB, Alföldi J, Wang Q *et al.* The mutational constraint spectrum quantified from variation in 141,456 humans. *Nature* 2020; **581**: 434–443.

4 Tan A, Abecasis GR, Kang HM. Unified representation of genetic variants. *Bioinformatics* 2015; **31**: 2202–2204.

5 Wang K, Li M, Hakonarson H. ANNOVAR: functional annotation of genetic variants from high-throughput sequencing data. *Nucleic Acids Res* 2010; **38**: e164.

6 Sherry ST, Ward MH, Kholodov M, Baker J, Phan L, Smigielski EM *et al.* dbSNP: the NCBI database of genetic variation. *Nucleic Acids Res* 2001; **29**: 308–311.

7 Sondka Z, Bamford S, Cole CG, Ward SA, Dunham I, Forbes SA. The COSMIC Cancer Gene Census: describing genetic dysfunction across all human cancers. *Nat Rev Cancer* 2018; **18**: 696–705.

8 Tong KI, Yoon S, Isaev K, Bakhtiari M, Lackraj T, He MY *et al.* Combined EZH2 Inhibition and IKAROS Degradation Leads to Enhanced Antitumor Activity in Diffuse Large B-cell Lymphoma. *Clin Cancer Res* 2021; **27**: 5401–5414.

9 Ross-Innes CS, Stark R, Teschendorff AE, Holmes KA, Ali HR, Dunning MJ *et al.* Differential oestrogen receptor binding is associated with clinical outcome in breast cancer. *Nature* 2012; **481**: 389–393.

10 Yu G, Wang L-G, He Q-Y. ChIPseeker: an R/Bioconductor package for ChIP peak annotation, comparison and visualization. *Bioinformatics* 2015; **31**: 2382–2383.

11 Heinz S, Benner C, Spann N, Bertolino E, Lin YC, Laslo P *et al.* Simple combinations of lineage-determining transcription factors prime cis-regulatory elements required for macrophage and B cell identities. *Mol Cell* 2010; **38**: 576–589.

12 Han H, Shim H, Shin D, Shim JE, Ko Y, Shin J *et al.* TRRUST: a reference database of human transcriptional regulatory interactions. *Sci Rep* 2015; **5**: 11432.

13 Kuleshov MV, Jones MR, Rouillard AD, Fernandez NF, Duan Q, Wang Z *et al.* Enrichr: a comprehensive gene set enrichment analysis web server 2016 update. *Nucleic Acids Res* 2016; **44**: W90–7.
